# A novel *LPL* intronic variant: g.18704C>A identified by re-sequencing Kuwaiti Arab samples is associated with high-density lipoprotein, very low-density lipoprotein and triglyceride lipid levels

**DOI:** 10.1371/journal.pone.0192617

**Published:** 2018-02-13

**Authors:** Suzanne A. Al-Bustan, Ahmad Al-Serri, Babitha G. Annice, Majed A. Alnaqeeb, Wafa Y. Al-Kandari, Mohammed Dashti

**Affiliations:** 1 Department of Biological Sciences, Faculty of Science, Kuwait University, Kuwait City, Kuwait; 2 Unit of Human Genetics, Department of Pathology, Faculty of Medicine, Kuwait University, Hawalli Governate, Kuwait; 3 Kuwait Medical Genetics Center, Ministry of Health, Kuwait City, Kuwait; University of Texas Health Science Center at San Antonio, UNITED STATES

## Abstract

The role interethnic genetic differences play in plasma lipid level variation across populations is a global health concern. Several genes involved in lipid metabolism and transport are strong candidates for the genetic association with lipid level variation especially lipoprotein lipase (*LPL*). The objective of this study was to re-sequence the full *LPL* gene in Kuwaiti Arabs, analyse the sequence variation and identify variants that could attribute to variation in plasma lipid levels for further genetic association. Samples (n = 100) of an Arab ethnic group from Kuwait were analysed for sequence variation by Sanger sequencing across the 30 Kb *LPL* gene and its flanking sequences. A total of 293 variants including 252 single nucleotide polymorphisms (SNPs) and 39 insertions/deletions (InDels) were identified among which 47 variants (32 SNPs and 15 InDels) were novel to Kuwaiti Arabs. This study is the first to report sequence data and analysis of frequencies of variants at the *LPL* gene locus in an Arab ethnic group with a novel “rare” variant (*LPL*:g.18704C>A) significantly associated to HDL (B = -0.181; 95% CI (-0.357, -0.006); p = 0.043), TG (B = 0.134; 95% CI (0.004–0.263); p = 0.044) and VLDL (B = 0.131; 95% CI (-0.001–0.263); p = 0.043) levels. Sequence variation in Kuwaiti Arabs was compared to other populations and was found to be similar with regards to the number of SNPs, InDels and distribution of the number of variants across the *LPL* gene locus and minor allele frequency (MAF). Moreover, comparison of the identified variants and their MAF with other reports provided a list of 46 potential variants across the *LPL* gene to be considered for future genetic association studies. The findings warrant further investigation into the association of g.18704C>A with lipid levels in other ethnic groups and with clinical manifestations of dyslipidemia.

## Introduction

Dyslipidemia or variation in plasma lipid levels is a global health concern that often leads to metabolic syndrome and subsequently heart disease. Although numerous environmental factors have been shown to increase the risk of hypertriglyceridemia (HTG) and hypercholesterolemia (HC), genetic factors remain unresolved. Different genetic studies on various populations have estimated the heritability values for plasma lipids to range between 40–60% [1–4]. These values are very likely to be influenced by ethnicity. Recent studies [3,5] reported that variation in triglyceride (TG), high density lipoprotein (HDL) and low-density lipoprotein (LDL) in addition to total cholesterol (TC) levels are influenced by specific variants at numerous gene loci that may contribute to the variation of these levels between different populations [1,6]. However, genetic association studies at various gene loci including Genome Wide Association Studies (GWAS) continue to report conflicting results and often reproducibility studies fail to confirm reported findings for specific genetic variants [2–6]. It has been documented that the main reason behind such conflicting results is the selection of variants (especially “rare” variants) that may be ethnic specific and therefore may not yield similar findings between different populations [3,5]. Nonetheless, genetic association of numerous variants at several gene loci involved in the lipid metabolic and transport pathways have been implicated, including lipoprotein lipase (*LPL*), to influence TG and HDL-C levels [3, 5].

*LPL* is an important rate-limiting enzyme for the hydrolysis of circulating TG, found in chylomicrons and VLDL, into non-esterified fatty acids and 2-monoacylglycerol for tissue utilization and HDL formation thereby regulating fatty acids [7, 8]. The 475 amino acids protein is encoded by a 30 Kb gene which has been mapped to chromosome 8p22 and has been fully sequenced [9]. The *LPL* gene consists of 10 exons with exons 1–9 averaging between 105–276 bp whereas exon 10 is much larger (1948bp) as it encodes the entire 3' untranslated region (UTR) [9]. The first exon has been shown to encode the 5' UTR, the signal peptide, and the first 2 amino acids of the mature protein [7]. The 8 exons in the middle encode the remaining 446 amino acids with exon 4 encoding the ApoC2 biding site [7].

Several studies have reported significant association between variants at the *LPL* gene locus and lipid parameters [3, 5, 6, 10–13]. In addition, sequence analysis of the *LPL* gene has revealed over one hundred single nucleotide polymorphisms (SNPs) within both the coding and noncoding region [8, 14–19]. Most variants that have been identified appear to have been based almost entirely on populations of European and/or African descent explaining <10% of the heritability factor on plasma lipid levels in the studied population [3, 5, 17, 18].

Sequence analysis studies involving both clinical lipid levels [6, 8, 15, 19, and 20] and interethnic genetic variation [17, 18] have demonstrated the importance of screening for mutations in order to establish association between plasma lipid levels and sequence variation specific to ethnic groups [3, 5]. Moreover, sequence analysis implicated numerous variants at the *LPL* gene locus with varying effect on TG and or HDL-C levels among different populations [8, 14–19, 21].

Recently, novel, “rare” and “common” variants at different regions of the *LPL* gene locus have been implicated in the increased risk of developing clinical HTG and or contributing to variable effects on either TG or HDL-C levels [8, 15–19]. In each of the studied population, at least 20 rare novel variants have been identified in groups with extreme TG or HDL-C levels [6, 15–19]. It was also reported that both TG and HDL-C levels may be variably affected in carriers of rare LPL variants [6, 19] and that the effect could be influenced by ethnicity [3, 5]. It has also been postulated that a cumulative effect of “rare” variants can increase the effect of a gene locus such as *LPL* on variation in plasma TG and HDL-C levels [1, 6, 19, 20]. In addition, “common” genetic variants of certain gene locus such as *LPL* may contribute to the missing heritability value of dyslipidemia [1, 6, 16] for different populations. Genetic association studies that analysed variants with regards to local ancestry reported interethnic variation in plasma lipid levels especially for TG and HDL-C levels where both global and local ancestries were cofounders for the association of SNPs in heterogeneous populations [3, 5].

The Kuwaiti population represents a heterogeneous group of Arabs with different ancestry [22] with the potential of identifying a variety of *LPL* gene variants and possible association with variation in plasma TG and HDL-C levels through resequencing the gene in a representative sample of Kuwaiti Arabs. There are limited studies that reported positive genetic association of common and rare variants with plasma lipid levels in the Kuwaiti population [23, 24]. The objective of this study was to 1) investigate sequence variation within the LPL gene locus in Kuwaiti Arabs, 2) compare the sequence and variants identified in Kuwaiti Arabs to that of other populations, 3) characterize and identify potential variants that could be associated with variation in plasma lipid levels and 4) conduct a genetic association study of novel variants with variation in plasma lipid levels in a cohort of the general native population.

The study is the first to report sequence data and analysis of the *LPL* gene locus in an Arab ethnic group with data that is comparable with those reported for non-Hispanic whites [17] and African Americans [18] in such a way that it may explain some of the observed plasma lipid levels in relation to interethnic variation with other ancestries.

## Methods

### Sample description and DNA extraction

A total of 100 Kuwaiti Arab DNA samples (50 males and 50 females) were selected from the DNA bank established at the Molecular Human Genetics lab in the Department of Biological Sciences at Kuwait University. Samples selected for inclusion were Arabs based on maternal and paternal lineages tracing back at least four generations as documented in their pedigrees.1 All selected participants were devoid of any metabolic diseases at the time of blood collection, and had documented lipid profiles and other relevant information such as BMI. Plasma lipid levels were determined at various clinical laboratories in both Mubarak Al-Kabeer and Al-Amiri hospital in Kuwait. Plasma lipid analysis included total cholesterol (TC), triglycerides (TG), High-density lipoprotein (HDL-C), Low-density Lipoprotein (LDL-C) all expressed in mmol/L. Reference values set by the Kuwait Ministry of Health for the Kuwaiti population were used to determine lower and higher percentile for TG and HDL-C levels. Demographic description of the samples (Table 1) and distribution of samples based on lipid levels (Fig A in S1 File). Validation of selected SNPs was carried out in an additional 702 randomly selected samples from the general Kuwaiti population including 282 males and 420 females (Table 1).

**Table 1:** Demographic data of the Kuwaiti Arab samples sequenced at the *LPL* gene locus analyzed for genetic association of 10 novel SNPs (B).

Variable	Age (yr)	BMI	TC (mmom/L)	TG (mmol/L)	VLDL (mmom/L)	HDL (mmom/L)	LDL (mmom/L)
**A. Kuwaiti Arabs (n = 100)**							
Range	18–70	19.26–29.7	2.8–7.7	0.24–2.03	0.1–0.81	0.64–1.42	1.7–6.3
Median	23	24.88	4.5	0.81	0.3427	1.06	3.1
Mean±SD	29.64±13.02	24.81±3.02	4.74±0.99	0.95±0.52	0.39±0.22	1.04±0.21	3.28±0.93
**B. Kuwaiti Cohort (n = 702)**							
Range	18–76	16.4–62.5	2–9.27	0.16–7.33	0.06–3.33	0.3–2.75	0.7–6.3
Median	28	25.7	4.6	0.83	0.34	1.1	3
Mean±SD	32.87±14.02	27.18±6.6	4.72±0.94	1.05±0.81	0.44±0.34	1.13±0.32	3.14±0.82

All participants in the study had given prior informed consent on the use of their samples in the current study. Ethical approval was obtained from the Kuwait Ministry of Health in accordance with the Helsinki guidelines of 1975. Total genomic DNA was extracted from all the blood samples using the salt extraction method described by Miller et al [25]. All DNA samples were analysed by nanodrop spectrophotometry and diluted to yield a final concentration of 25ng/μl for the sequencing and Real-time PCR reactions.

### *LPL* re-sequencing: Primer design and amplification protocol

Based on the complete published human reference sequence in GenBank (http://www.ncbi.nlm.nih.gov, NCBI) 74 different primer sets flanking a 700bp region throughout the *LPL* gene (30Kb gene locus and 1Kb downstream and upstream of the gene) were designed using Primer 3 (v. 0.4.0) and found to be reproducible and consistent in generating overlapping products for the sequencing of the full gene as they covered the whole target sequence from nucleotide position 19939071–19967259. The 74/2 primer sequences, their location, product size and annealing temperatures are provided (Table A in S2 File). Amplification conditions for the polymerase chain reaction (PCR) with the designed primers employed for the amplification of the target sequences across the *LPL* gene locus in 100 Kuwaiti samples of documented Arab ethnicity is provided (Tables B and C in S2 File). PCR products were purified by Nucleospin® Gel and PCR Clean-up Kit (Macherey-Nagel 740609.250) and sequenced using the BigDye™ Terminator v3.1 Cycle Sequencing Kit (Applied Biosystems, 4337455) for the 74 regions starting at the 3’ end in separate reactions with the forward primer only. The products were then subjected to denaturation of the double stranded DNA using a BigDye XTerminator™ Purification kit (Applied Biosystems, 4376486). The samples were sequenced on the ABI 3130xl genetic analyser and the sequence data was analysed by the ABI DNA sequencing Analysis Software (version 2.5). For quality assurance of the sequence data generated, a separate sequencing reaction was performed using a reverse primer on all the samples following the same steps as the forward primer.

### *LPL* sequence variation analysis in Kuwaiti Arabs

Variants, including both SNPs and InDels, were identified based on those reported at the *LPL* gene locus and genome assembly GRCh38 [26]. The 2,269 variants published in the NCBI database, which were assigned reference numbers at the *LPL* Gene were used for confirmation of the identified variants. The newly created database file for the Kuwait LPL data was used to compare reported variants and identify novel variants in the data generated from the 100 Kuwaiti Arab samples. Characterization of the novel SNPs and InDels were performed using Ensembl Variant Effect Predictor tool based on gene build 89 and genome assembly GRCh38 [26]. To identify potential variants for association analysis of LPL with variation in lipid levels, the minor allele frequency of 254 identified variants were estimated for HTG, low TG, high HDL-C and low HDL-C, separately, using the GenABEL package [27] for R software version 3.2.2 [28]. The minor allele frequency differences were calculated for each of these SNPs among high and low TG populations, and high and low HDL-C populations.

### Validation and genetic association analysis of selected identified novel *LPL* SNPs

Of the 47 novel variants identified, 11 SNPs were selected for validation and genetic association analysis with regards to lipid levels based on the following criteria: 1) variants that had a minor allele frequency (MAF) ≥0.1%, 2) those with In Silico predicted functional significance (the 3’UTR– 44), 3) variants representing the different regions across the *LPL* gene locus and 4) those that were at either extreme of the TG levels. InDels were excluded from the selection criteria as there were challenges in designing Real-Time PCR probes.

Real-Time PCR was used for the validation of the 11 selected novel variants in the 702 Kuwaiti samples using the ABI 7900HT Fast Real-Time PCR (Applied Biosystems, GS 01/02). Custom primers and probes targeting the novel SNPs identified were designed for genotyping the cohort samples. Genotyping was achieved with the allele discrimination assay and carried out using the Real-Time PCR software (Applied Biosystems). Genotyping the samples as homozygous or heterozygous for each SNP were relatively simple to establish for reporting by the SDS software. Details of all the above methods are provided in my protocols (dx.doi.org/10.17504/protocols.io.mhcc32w).

### Statistical analysis

Allele and genotype frequencies were determined by the simple gene-counting method for all the variants identified at the *LPL* gene locus in the 100 samples of Kuwait Arabs sequenced. Deviation from Hardy-Weinberg equilibrium (HWE) was investigated with GENEPOP [29] software (Version 4.2) at a significance level of p> 0.05. Subject characteristics including plasma lipid levels are expressed as mean±Standard Deviation, median, and range (Table 1).

The selected 7 variants validated with Real-Time PCR were further analysed for HWE in the cohort (n = 702). The SNPs with a minor allele frequency of MAF≥0.1% and that were in HWE were further investigated. LPL novel SNPs and lipid levels were evaluated by the Kruskal-Wallis and the Mann–Whitney tests as appropriate. Significant and borderline SNPs were further analysed by a multivariate analysis using linear regression represented as beta (B) coefficient and 95% confidence intervals (CI). Age and gender were controlled for in the linear regression and significance was set at p < 0.05. Analysis was performed using SPSS software (version 23; SPSS Inc, Chicago, IL, USA).

## Results

### Re-sequencing of the *LPL* gene of Kuwaiti Arabs

The target regions at the *LPL* gene locus were successfully sequenced, aligned, analysed and screened for variants in all 100 Kuwaiti Arab DNA samples. The verified complete sequences obtained from overlapping regions using the 74 newly designed primers were aligned and compared with the published reference sequence (NG_008855.1) in the GenBank database using the AB Seqscape software (Version number 2.5). The sequenced region (33,755bp) of the *LPL* sequence including 3’ and 5’ flanking sequences (chromosome 8: 19,967,825–19,934,070) were deposited in GENBANK and assigned the accession numbers *LPL* KU557518 (BankIt1887509) and KY448281 for the SNPs submissions.

### *LPL* sequence variation analysis in Kuwaiti Arabs

**Fig 1:**
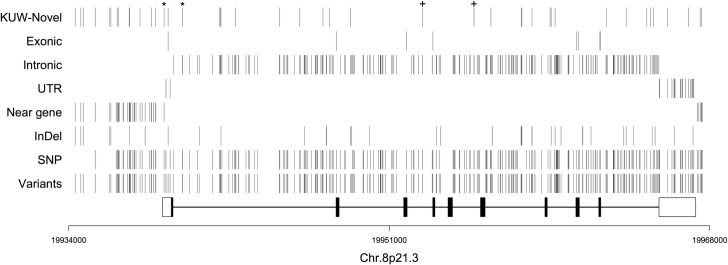
Distribution of the total variants (n = 291) including the 47 novels identified at the *LPL* gene locus among the 100 Kuwaiti Arab samples. The variants are shown based on their location across the 30Kb gene. The shaded boxes represent exons 1–9 and the open box represents the untranslated regions (UTR). The positions of the variants are based on gene build 89 and genome assembly GRCh38 [26]. The (+) indicate the novel variants KUA LPL-27 and KUA LPL-28. The stars indicate the variants (KUA LPL-15 and KUA LPL-17) selected for validation yet failed synthesis by real-time PCR.

A summary of the number and type of SNPs, including novel SNPs, identified from the regions is provided (Table D in S2 File). Numerous variants (n = 293) were identified in both the coding and non-coding regions of the gene including novel SNPs and InDels (Fig 1). However, no novel variants were identified in the coding regions. The identified SNPs and InDels at the *LPL* gene locus included 47 novel and 246 previously reported variants. Analysis of the newly identified variants with regards to their location within the gene, classification and frequency was also determined (Table 2). There were 15 InDels, 6 of which were in the 5' flanking sequence. Of the remaining 9, 3 were found in intron 6, 2 in intron 7, and 1 each in exon (5'UTR) 1, introns 1, 2, and 9. These were mostly insertions of 1–4 nucleotides and one deletion of a single nucleotide in intron 1. All InDels were found to be heterozygous with an MAF of ≥1% (n = 100). Two Indels (KUA LPL-26 and KUA LPL-36) were found in a heterozygous state for all the samples sequenced (n = 100) and were found to deviate from HWE (Table 2). KUA LPL-26 involved a single nucleotide (A) insertion at position 14865 of intron 2 while KUA LPL-36 involved 3 nucleotide (TTT) insertions between positions 25730–25732 of intron 7.

**Table 2:** A summary of the 47 novel variants identified by re-sequencing the full *LPL* gene locus and flanking sequences based on genome assembly GRch38.p10 [26].

	Variants*	Chromosome Location	Position in gene	Consequence
1	**KUA LPL-1**	8:19934341–19934342	5’ Near gene	upstream_gene_variant
2	KUA LPL-2	8:19934643–19934643	5’ Near gene	upstream_gene_variant
3	**KUA LPL-3**	8:19934791–19934792	5’ Near gene	upstream_gene_variant
4	KUA LPL-4	8:19935408–19935408	5’ Near gene	upstream_gene_variant
5	**KUA LPL-5**	8:19936157–19936158	5’ Near gene	upstream_gene_variant
6	**KUA LPL-6**	8:19936234–19936235	5’ Near gene	upstream_gene_variant
7	KUA LPL-7	8:19936578–19936578	5’ Near gene	upstream_gene_variant
8	KUA LPL-8	8:19936636–19936636	5’ Near gene	upstream_gene_variant
9	KUA LPL-9*	8:19937211–19937211	5’ Near gene	upstream_gene_variant
10	**KUA LPL-10**	8:19937232–19937233	5’ Near gene	upstream_gene_variant
11	KUA LPL-11	8:19937274–19937274	5’ Near gene	upstream_gene_variant
12	KUA LPL-12	8:19937783–19937783	5’ Near gene	upstream_gene_variant
13	KUA LPL-13	8:19938389–19938389	5’ Near gene	upstream_gene_variant
14	KUA LPL-14	8:19938609–19938609	5’ Near gene	upstream_gene_variant
15	KUA LPL-15	8:19939059–19939059	5’ UTR	5_prime_UTR_variant
16	**KUA LPL-16**	8:19939272–19939272	Exon 1	non_coding_transcript_exon_variant
17	KUA LPL-17	8:19940038–19940038	Intron 1	intron_variant
18	KUA LPL-18	8:19941997–19941997	Intron 1	intron_variant
19	KUA LPL-19	8:19942062–19942062	Intron 1	intron_variant
20	**KUA LPL-20**	8:19942076–19942077	Intron 1	intron_variant
21	KUA LPL-21	8:19942844–19942844	Intron 1	intron_variant
22	KUA LPL-22	8:19945173–19945173	Intron 1	intron_variant
23	KUA LPL-23	8:19946250–19946250	Intron 1	intron_variant
24	KUA LPL-24	8:19947485–19947485	Intron 1	intron_variant
25	KUA LPL-25	8:19947845–19947845	Intron 1	intron_variant
26	KUA LPL-26*	8:19948936–19948936	Intron 2	intron_variant
27	KUA LPL-27	8:19952774–19952774	Intron 3	intron_variant
28	KUA LPL-28	8:19955496–19955496	Intron 5	intron_variant
29	KUA LPL-29	8:19955498–19955498	Intron 5	intron_variant
30	KUA LPL-30	8:19956392–19956392	Intron 6	intron_variant
31	**KUA LPL-31**	8:19958004–19958004	Intron 6	intron_variant
32	**KUA LPL-32**	8:19958036–19958036	Intron 6	intron_variant
33	**KUA LPL-33**	8:19958575–19958575	Intron 6	intron_variant
34	KUA LPL-34	8:19959535–19959535	Intron 7	intron_variant
35	**KUA LPL-35**	8:19959593–19959593	Intron 7	intron_variant
36	KUA LPL-36*	8:19959799–19959799	Intron 7	intron_variant
37	KUA LPL-37	8:19959940–19959940	Intron 7	intron_variant
38	KUA LPL-38	8:19962529–19962529	Intron 9	intron_variant, regulatory _ region _variant
39	**KUA LPL-39**	8:19963567–19963568	Intron 9	intron_variant
40	KUA LPL-40	8:19963891–19963891	Intron 9	intron_variant
41	KUA LPL-41	8:19964662–19964662	Intron 9	intron_variant
42	KUA LPL-42	8:19964869–19964869	Intron 9	intron_variant
43	KUA LPL-43	8:19965014–19965014	Intron 9	intron_variant
44	KUA LPL-44	8:19965760–19965760	3’UTR	3_prime_UTR_variant
45	KUA LPL-45	8:19966866–19966866	3’UTR	3_prime_UTR_variant
46	KUA LPL-46	8:19967489–19967489	3’ near gene	downstream_gene_variant
47	KUA LPL-47	8:19967551–19967551	3’ near gene	downstream_gene_variant

Highlighted variants are InDels

* Variant frequency deviated from Hardy-Weingberg Equilibrium (HWE)

Consequence of the variants on the *LPL* gene was predicted by Ensembl Variant Effect Predictor tool [26].

The 32 SNPs identified included 9 in the 5’ flanking sequence and 2 in the 3’ flanking sequence. The remaining 21 novel variants were SNPs localized within the *LPL* gene and included 47.62% (n = 10) transitions of which 5 were C to T and 3 G to A and 52.38% (n = 11) transversions of which 5 were G to C. Only two of these SNPs (KUA LPL- 28 and KUA LPL- 40) had an MAF ≥ 1% and were selected for validation in the cohort. The remaining 16 SNPs had an MAF<1% and were found in a single heterozygous state among the sampled individuals.

**Fig 2:**
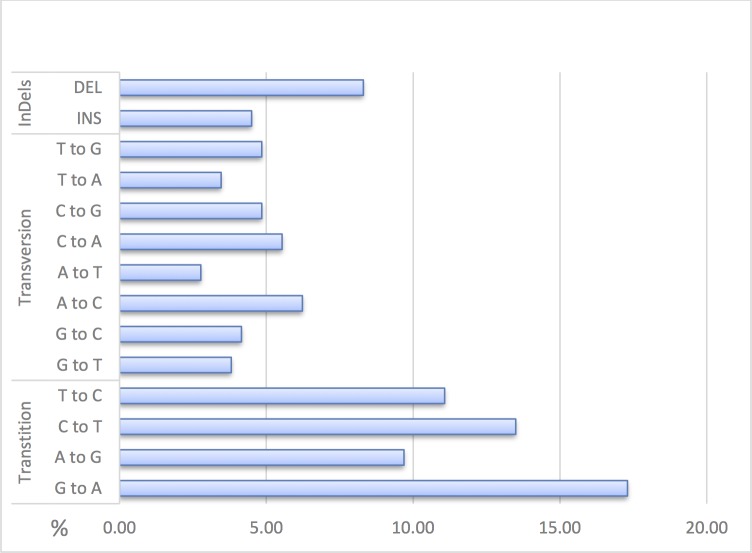
Distribution (represented in percentage) of the type of variants identified across the *LPL* gene locus in the Kuwaiti Arabs (n = 100).

The highest number of identified variants were found in the intronic regions (n = 219 out of 293) (Fig B in S1 File). The total variants identified across the *LPL* gene locus included 37 InDels (with a range of 1 to 4 nucleotides) and 252 SNPs consisting of 149 transitions representing 51.56% of the total variants followed by 35.64% transversions and 12.68% InDels (Fig 2). The highest frequency of variants was identified in intron 1 and the lowest in intron 4 (Fig B in S1 File). Similarly, most InDels were found in the intronic regions (74%) followed by the 3’ (5%) and 5’UTR (3%). Most SNPs were base pair substitutions and localized to the non-coding regions except for 7 variants identified in the protein coding regions. These included 3 silent base-pair substitutions in exon 2 (rs1801177), 3 (rs1121923), 4 (rs248), 2 SNPs in exon 8 (synonymous variant rs316 and a missense variants rs5934) and 2 in exon 9 (missense variant rs150319057 and the stop-gain variant rs328). A total of 118 common variants (MAF>5%), 57 rare variants (MAF<5%) and 118 very rare variants (MAF<1%) have been identified throughout the *LPL* locus (Fig C in S1 File). The distribution of the variants amongst the four groups analysed based on their TG and HDL-C levels were observed was variable (Table E in S2 File).

### Analysis and characterization of *LPL* novel variants

Ensembl Variant Effect Predictor (VEP) tool, based on genome assembly GRCh38 [26], allowed the annotation and comparison of the reported variants at the *LPL* gene locus with 1,296 of previously published variants across the *LPL* gene locus. The effect of the total variants (n = 291) identified in this study are summarized (Table F in S3 File). To identify potential *LPL* variants association with variation in lipid levels, the differences in the minor allele frequency of the 254 identified and previously reported variants were estimated for HTG, low TG, high HDL-C and low HDL-C, separately. A total of 47 SNPs were found to have a frequency difference of ±0.075 between low and high TG and 37 SNPs between low and high HDL-C (Figs E and F in S1 File).

Most of the novel variants predicted consequence does not have high impact on the protein function based on the Ensembl VEP tool gene build version 89 genome assembly GRCh38 (Table B in S2 File). However, one variant (3’UTR-Novel SNP 44) was predicted to have a functional significance related to miRNA binding (has-miR-3648 binding site) as predicted by the software MR SNP specialized for the functional analysis SNPs in the 3'UTR [30]. This SNP was found to have a difference in its genotype distribution at either extreme of the TG and/or HDL-C levels based on the analysis of the differences in allele frequencies (Fig E in S1 File). This SNP and others like it were considered for validation. Along with this SNP, 13 SNPs that were associated with either extreme TG levels and 16 for HDL levels were selected for validation. From these, two (KUA LPL-27 and 28) were found at the extremes for both TG and HDL levels and had an MAF≥0.1%. In addition, 9 SNPs from both groups of high and low TG/HDL levels and based on the criteria described in methods were selected for further analysis with regards to potential association with TG and HDL-C levels in the cohort of 702 samples obtained from the general Kuwaiti population (Table 3).

**Table 3:** Selected LPL novel variants, their characteristics and validation in the Kuwaiti cohort.

SNP	Chromosomal Position	Gene Location	Predicted Function	Marker Name	MAF* (N = 702)
KUA LPL-15 ss2137497746	8:19939059G/A	5' near gene	5’ UTR variant	Failed Synthesis based on QA	-
KUA LPL-17 ss2137497747	8:19940038G/C	Intron 1	Intron variant	Failed Synthesis based on QA	-
KUA LPL-22 ss2137497748	8:19945173G/C	Intron 1	Intron variant	AHD2DCZ	C:0.001
KUA LPL-27 ss2137497749	8:19952774C/A	Intron 3	Intron variant	AHFBBI7	A:0.009
KUA LPL-28 ss2137497750	8:19955496C/T	Intron 5	Intron variant	AHGJ9PF	T:0.011
KUA LPL-30 ss2137497751	8:19956392T/C	Intron 6	Intron variant	AHHS7VN	C:0.001
KUA LPL-37	8:19959940C/G	Intron 7	Intron variant	AHI151V	0.000
KUA LPL-38 ss2137497752	8:19962529C/T	Intron 9	Intron variant regulatory variant	AHKA373	T:0.002
KUA LPL-40	8:19963891C/G	Intron 9	Intron variant	AHLJ2EB	0.000
KUA LPL-44 ss2137497753	8:19965760C/T	3'UTR	3’ UTR variant	AHMS0KJ	T:0.003
KUA LPL-45 ss2137497754	8:19966866 C/A	3'UTR	3’ UTR variant	AHN1YQR	A:0.001

MAF: Minor Allele Frequency.

* All SNPS were in HWE (p>0.05).

Based on genome assembly GRCh38 [26].

### Validation and genetic association analysis of selected identified novel *LPL* SNPs

A sample of the Real-time PCR allelic discrimination plot for the novel variants is provided (Fig F in S1 File). Two SNPs failed amplification by Real-time PCR and therefore were excluded from further analysis. In addition, two other SNPs were also excluded since they were not detected in any additional samples of the cohort. Analysis of the remaining 7 novel variants (Table 3) with regards to their genotype distribution among the cohort showed a frequency of less than 1% for five of the novel SNPs and two were higher than 1%, all of which were found in HWE. Univariate analysis revealed a significant association between KUA LPL-27 and low HDL levels (Table 4) confirming the preliminary finding observed (Fig E in S1 File).

**Table 4:** Genetic association of the two novel *LPL* variants with plasma lipid levels among the Kuwaiti cohort.

	KUA LPL-27 Genotypes ss2137497749			KUA LPL-28 Genotypes ss2137497750		
Variable*	CC (n = 689)	CA (n = 13)	P-value	CC (n = 683)	CT (n = 16)	P-value
HDL	1.13±0.32	0.93±0.25	0.033	1.13±0.32	1.1±0.3	0.863
TC	4.73±0.94	4.35±0.94	0.303	4.72±0.95	4.64±0.88	0.645
TG	1.05±0.81	1.35±0.81	0.059	1.05±0.81	1.29±0.9	0.155
VLDL	0.44±0.35	0.55±0.31	0.064	0.44±0.34	0.52±0.3	0.16
LDL	3.12±0.81	2.74±0.86	0.242	3.1±0.81	2.98±0.98	0.291

*Plasma Lipid Levels are expressed as mmol/L and standard deviation.

Multivariate analysis revealed that the minor allele of this SNP remained significant for association with HDL-C levels (B = -0.181; 95% CI -0.357, -0.006); 0.043) after adjusting for age and sex. In addition, a trend with the novel variant was observed for TG as well as VLDL levels, however, the p-value was >0.05 (Table 5). Linear regression was conducted to analyse the association between the novel SNP and both traits TG and VLDL independently along with adjusting for both age and gender with results showing a borderline significance p = 0.044 and 0.043 respectively (Table 5).

**Table 5:** Multivariate analysis using linear regression to predict the effect of KUA LPL-27 genotypes ss2137497749 on HDL, TG and VLDL levels in the Kuwaiti cohort (n = 702).

Variable	B coefficient and 95% CI	P-value
**HDL**		
Age	-0.002 (-0.004, -0.001)	0.008
Sex	-0.255 (-0.3, -0.21)	0.0001
*LPL* novel SNP	-0.181 (-0.357, -0.006)	0.043
**TG**		
Age	0.009 (0.007–0.01)	<0.0001
Sex	0.109 (0.073–1.45)	<0.0001
*LPL* novel SNP	0.134 (0.004–0.263)	0.044
**VLDL**		
Age	0.009 (0.008–0.01)	<0.0001
Sex	0.107 (0.7–0.143)	<0.0001
*LPL* novel SNP	0.131 (-0.001–0.263)	0.043

## Discussion

### Analysis of LPL sequence variation in Kuwaiti Arabs

**Fig 3:**
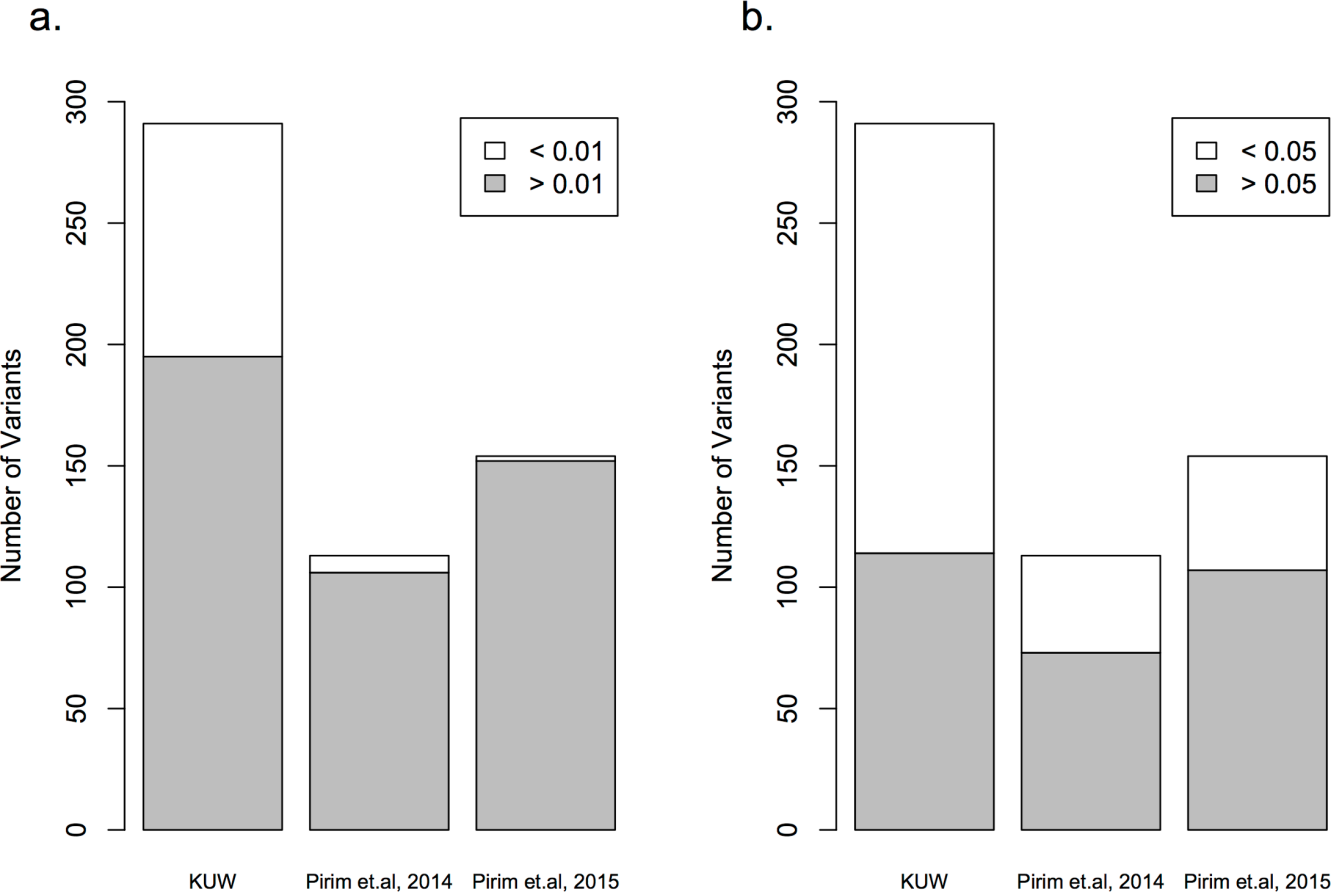
Comparison of the variants based on the minor allele frequencies (MAF) identified across the *LPL* gene locus in different populations including the Kuwaiti Arab samples from this study.

**Fig 4:**
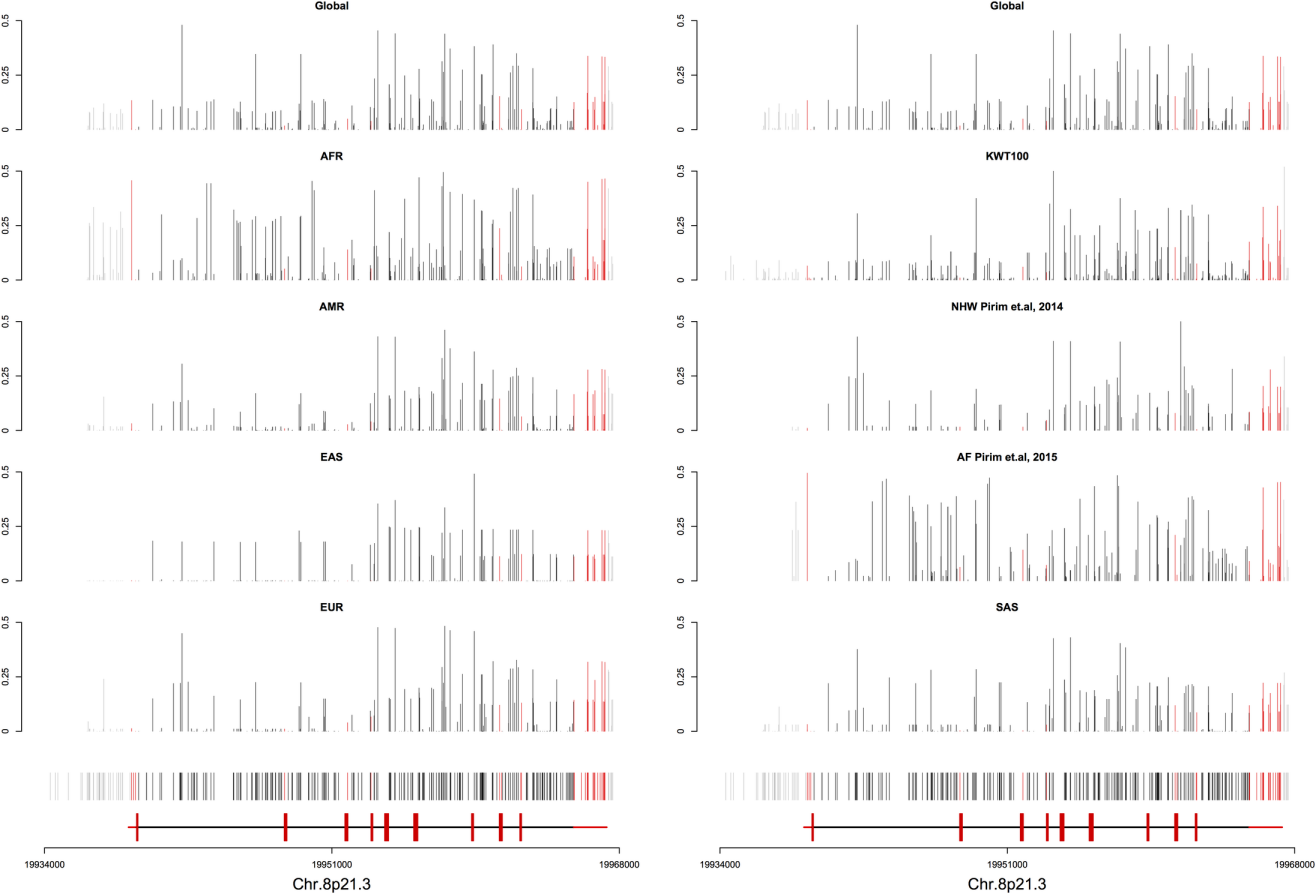
Comparison of the frequencies of rare (a) and common (b) variants identified at the *LPL* gene locus in the Kuwaiti Arab samples (n = 100), non-Hispanic whites (n = 95; Pirim et al., 2014) and American Africans (n = 95; Pirim et al., 2015). The variants identified in this study are shown based on their location across the 30Kb gene. The minor allele frequencies are based on gene build 89 and genome assembly GRCh38 [26].

A total of 293 variants were identified, 84% of which were previously reported with variable frequencies across different populations. Previous studies that re-sequenced the full gene in other populations reported similar variability at the *LPL* gene locus in which most variants were located in the non-coding regions of the gene. A comparison between the sequence data analysis of the Kuwaiti Arab population with both non-Hispanic whites (NHW) in which a total of 176 variants were identified in 95 samples [17] and African Americans (AA) in which a total of 308 variants were identified in 95 samples [18] confirmed diverse sequence variants [14] (Fig 3). The distribution of variants across the *LPL* gene in the Kuwaiti Arab ethnic group was found to be like other populations where the highest variability was in the intronic regions in which the number of variants reflected the size of the introns (Fig 4). In addition, the number of novel variants identified (28 variants) in NHW was the lowest in comparison to AA (64 variants) and Kuwaiti Arabs (47 variants). Based on the number of variants found, their distribution and MAF, the diversity of the *LPL* gene in Kuwait Arabs falls between that of African and Caucasian populations.

Nickerson et al. [14] have reported that the average nucleotide diversity at the *LPL* gene locus is 0.2% reflecting one change per 500bp with different site variation across different populations supporting the high variability observed in the Kuwaiti Arabs. In addition, the analysis in this study showed differences in the average MAF across different populations (Table F in S3 File). The MAF for “common”, “rare” and “very rare” alleles can also be variable between different populations (Fig 3). The number of variants identified in the Kuwaiti Arabs (n = 100) for “common” and “rare” was found to lie between those reported for NHW [17] and AA [18]. However, the higher number of 118 “very rare” variants observed in this study as compared to that of 36 in NHW [17] and 60 in AA [18] may very likely be an outcome of having sequenced a wider region of the flanking sequences which yielded 41 variants of the total 293. Observed differences in MAF of variants across the *LPL* gene locus between the studied and compared populations strongly suggests the need to be selective of variants for specific ethnic groups in genetic association studies.

Another important point to consider is that the higher diversity in introns (n = 219) identified in a small sample (n = 100) may be misleading especially in genetic association studies as to their role in disease manifestation or if they are simple variation in a specific population since these sequences don’t code for the polypeptide chain. Numerous variants (Fig B in S1 File) in the introns of the *LPL* gene have been reported with positive association with TG and/or HDL-C levels [3, 5, 6, 17, and 18]. In our study, 75% of the variants were identified in introns with more than 10% of the total in introns 1 (n = 58), 6 (n = 37), 9 (n = 37) and 2 (n = 26) and less than 10% in introns 7 (n = 19), 5 (n = 17), 8 (n = 13), 3 (n = 8) and 4 (n = 4). This is in agreement with the findings of Pirim et al. [18] in which the highest variation was observed in introns 1, 6 and 9. These results may be a reflection of the actual size of the introns since the largest intron at the *LPL* gene is intron 1 (8,651bp) followed by introns 2 (3,428bp), 6 (3,176bp) and 9 (3,090) (Table H in S2 File). The average number of novel variants in the intronic regions reported in NHW (n = 24; 92%) [17] was similar to that observed in Kuwaiti Arabs (n = 27; 87%) and much lower than AA (n = 51; 85%) [18] after excluding the variants of the flanking regions.

Since dyslipidemia is multifactorial and may be influenced by variants located in the intronic regions and since many studies on Arabs have shown association with ethnicity [23, 24], it becomes imperative that ethnicity and ancestral background be considered when conducting genetic association studies.

### Genetic association of novel *LPL* variants with variation in plasma lipid levels

Recent studies have documented the importance of considering local ancestry when estimating the effect size of candidate genetic variants, including *LPL*, on variation in lipid levels [3, 5].

In fact, it has been reported that variation in lipid levels between individuals and populations of different ethnic groups may be highly attributable to genetic variants explaining observed differences in the range of normal plasma lipid values [1, 3, and 5].

Most of these variants lie in the noncoding regions of the *LPL* gene. The functional importance of variants in introns of the *LPL* gene, like for many genes, remains unresolved despite numerous studies indicating their role in regulation, splicing and epistasis. The main objectives of this study were to unveil novel variants that could be specific to Arab ethnic groups predisposing them to some form of dyslipidemia. From the total number of novel variants identified in Kuwaiti Arabs (n = 47), 16 were found in the flanking regions, 27 in the intronic regions and only 3 in the UTR of the *LPL* gene locus. Of these, three were not in HWE (Table 2) and therefore were excluded from any further analysis. Analysis of the 32 remaining novel SNPs (Fig E in S1 File) allowed the selection of candidates for genetic association with TG and HDL-C levels. Only one SNP (KUA LPL-27: g.18704C>A) was found to be significantly (p = 0.043) associated with low HDL-C levels in the Kuwaiti cohort (Table H in S2 File). This may indicate that the minor allele for this SNP is not a mere population variant but may have a functional significance in increasing the risk to dyslipidemia. Another interesting observation with regards to this SNP is that it also displayed a trend for association (Table H in S2 File) with TG levels (Fig E in S1 File). This was supported by multivariate analysis which revealed a significant (p<0.05) increase in both TG and VLDL levels in carriers of this SNP while having a significant decrease (p<0.05) in HDL levels. Though these lipoproteins are involved in different lipid transport pathways, variation in their levels lead to similar consequences. Ensembl predictor effect analysis of this novel variant indicated that it is just an intron variant similar to those variants in intron 3. However, it may be argued that this SNP is within an important regulatory region of the *LPL* gene as it lies between coding sequences for the ApoC2 binding site (exon 4) and amino acids sequence important for the catalytic activity of LPL [7]. In addition, this variant showed a significant association on opposing effects of TG, VLDl to HDL levels that may be explained by a possible direct-action on TG and VLDL levels and an indirect action on HDL levels. The catalytic activity of LPL is directly related to TG and subsequently VLDL and this variant maybe affecting the splicing mechanism which compromises the protein structure and function [7]. However, it can be postulated that the same variant is influencing lower levels of HDL through an indirect mechanism. It may be interacting either with other variants reflecting its potential "modifying" role on gene expression needed for the reverse cholesterol transport pathway or by facilitating binding of transcription factors at other gene loci involved in the pathway [31]. It has also been reported that HDL-C levels can be influenced by LPL activity through production of TG-rich lipoproteins remnants [6]. Numerous studies have reported an association of adjacent SNPs in intron 3 with variation in HDL-C and/or TG levels such as rs343 and rs75026342 [3, 6]. SNP rs343 (+13836C>A) was reported to be a likely disease marker for type 2 diabetes mellitus in the Chinese population [31].

KUA LPL-27 was found to be associated with variation in HDL-C levels as well as TG and VLDL levels and as such it is strongly suggested that it be tested in other ethnic groups and populations for similar effects. To the best of our review of literature, limited studies have documented a positive genetic association between *LPL* and VLDL levels [11]. Functional predication for the action of this variant may be to have a regulatory role in splicing [19, 31] or in modulating the binding of the transcription factor [32]. It is probable that the effect of this variant lies in its disruption of the intron's organization role in the splicing of exon 4. In turn, altered splicing may therefore affect the catalytic activity of LPL leading to accumulation of circulating TG and VLDL (Table 5). This needs to be investigated but would require analysis of mRNA and LPL protein levels. Other studies have reported that novel pathogenic variants occurring in introns, such as intron 2 and 6, may lead to the production of truncated proteins rendering LPL from its activity [19]. Our findings support the need for accurately identifying sites of variation and the effect they might have on the expressed protein product through correlating and analysing their effect on plasma lipid levels. This would allow us to design better genetic association studies for specific ethnic groups [3, 5, 21] and unveiling molecular mechanisms regulating plasma lipid levels.

#### Potential of other *LPL* variants for genetic association with variation in plasma lipid levels

Another objective of our study was to identify “common”, “rare” and “very rare” variants that would serve as potential markers in future genetic association studies of dyslipidemia in Arabs. Of the 252 SNPs identified, those showing allelic differences between two extremes of TG and\or HDL-C levels by a frequency difference of more than 0.1 and reported previously for their genetic association have been reviewed (Table 6 and Table G in S2 File). Of these, a total of 46 potential variants (only 1 InDel rs252) across the *LPL* gene locus were compared to other re-sequencing studies involving individuals free of clinical diseases [16–18] and involving the effect of local ancestry [3, 5] in which there was a potential indicator of TG and/or HDL-C level variation. Of these, 14 displayed a potential for exerting opposite effects on TG and HDL-C levels in the Kuwaiti Arab re-sequenced sample. Nine variants showed differences in allelic frequencies at the lower TG extreme and for the high HDL-C extreme suggesting a possible protective role against dyslipidemia. In addition, few implicated rare variants in the coding regions and in splice junctions of the *LPL* gene have been previously reported by Evans et al. [15] where at least 20 rare variants were involved in variation of TG and HDL-C levels. However, of the three variants identified (rs248, rs316 and rs1801177), only rs248 was found to have a difference between the two TG and HDL-C opposite extremes in this study.

**Table 6:** List of 46 potential SNPs at the *LPL* gene locus for genetic association studies of dyslipidemia, the metabolic syndrome and coronary heart disease.

SNP	Position	KMAF (n = 100)	GMAF	Reported a significant association with TG and or HDL-C	Reference
rs1801177	14127G>A	0.01	0.0176	Iranians AF NHW Spaniards	Askari et al., 2016 [12] Ariza et al., 2010 [10] Pirim et al., 2015 [18] Pirim et al., 2014 [17] Evans et al., 2011 [15] Wood et al., 2011 [11]
rs34123038	15574G>A	0.065	0.0212	Demonstrates opposite effect on TG compared to HDL-C	
rs74304285	16449G>A	0.085	0.1396	Demonstrates opposite effect on) compared to HDL-C Reported association with TG (p<0.01)	Bentley et al., 2014 [5]
rs8176337	15090C>G	0.25	0.346	Both with TG and HDL-C	Pirim et al., 2015 [18]
rs248	19245G>A	0.035	0.0387	Never been tested	Pirim et al., 2014 [17] Evans et al., 2011 [15]
rs252	19651delA	0.5	0.4543	A novel association with LDL-C	Pirim et al., 2015 [18]
rs263	21231 C>T	0.205	0.2476	In proximity with KUA LPL-28	
rs281	23442A>T	0.17	0.3129	Demonstrates opposite effect on TG compared to HDL-C	
rs283	23517C>T	0.135	0.264	Opposite effect on TG compared to HDL-C. Chinese obese adolescents with the GG genotype were more sensitive to exercise-induced reduction TG level	Goa et al., 2015 [33]
rs295	24657 A>C	0.23	0.2746	Association with TG ad HDL-C	Pirim et al., 2014 [17]
rs294	24544 T>C	0.12	0.1322	First tested	Pirim et al., 2014 [17]
rs282	23445C>G	0.095	0.0585	First tested	Pirim et al., 2014 [17]
rs277	22822T>C	0.05	0.1378	First tested	Pirim et al., 2014 [17]
rs279	23115C>G	0.02	0.0387	Association with TG ad HDL-C	Pirim et al., 2015 [18]
rs286	23675 A>T	0.06	0.0331	Association with TG ad HDL-C	Pirim et al., 2014 [17]
rs301	25353T>C	0.225	0.382	Demonstrates opposite effect on TG compared to HDL-C	
rs304	25780T>G	0.235	0.2532	Demonstrates opposite effect on TG compared to HDL-C	
rs305	25820A>G	0.235	0.2526	Demonstrates opposite effect on TG compared to HDL-C	
rs327	27955T>G	0.29	0.2925	Association with TG and HDL-C	Smith et al., 2010 [34]
rs316	26855C>A	0.15	0.1526	Association with TG and HDL-C	Pirim et al., 2014 [17] Evans et al., 2011 [15]
rs320	32067 A>T	0.335	0.3375	Association with TG and HDL-C	Askari et al., 2016 [12] Pirim et al., 2014 [17] Ariza et al., 2010 [10]
rs328	28143C>G	0.075	0.0925	Associated with TG level between Chinese ischemic stroke patients and controls Association with HDL-C and TG in Asian population Reported effect size on TG regardless of ethnicity Reported an association with plasma HDL-C levels	Yue et al., 2017 [35] Shahid et al., 2017 [13] Ayyappa et al.,2017 [36] Askari et al., 2016 [12] Pirim et al., 2014 [17] Evans et al., 2011 [15] Deo et al., 2009 [3] Bentley et al., 2014 [5] Ariza et al., 2010[10] Lu et al., 2008[36] Ayyappa et al., 2017 Askari et al., 2016 Pirim et al., 2014 Evans et al., 2011 Deo et al., 2009 Bentley et al., 2014 Ariza et al., 2010 Lu et al., 2008
rs329	28505A>G	0.04	0.0369	Association with TG and HDL-C	Pirim et al., 2015[18]
rs12679834	28852T>C	0.09	0.0972	Reported effect size on TG regardless of ethnicity; Reported Significance (p = 0.0009) with TG	Deo et al., 2009 [3] Pirim et al.; 2014 & 2015[17, 18] Bentley et al., 2014
rs3208305	32067 A>T	0.335	0.3375	Reported significance (p = 0.0001) with TG. Reported significant association with plasma HDL-C levels)	Evans et al., 2013 [16] Lu et al., 2008 [37]
rs1803924	32093 C>T	0.09	0.0913	Reported significance (p = 0.019) with TG	Evans et al., 2013 [16]
rs3735964	32464 C>A	0.085	0.0903	Reported significance (p = 0.006) with TG	Evans et al., 2013 [16]
rs3200218	32490A>G	0.08	0.1488	reported significance (p = 0.0769) with TG and loss of miR-489	Evans et al., 2013[16]
rs13702	32911 T>C	0.34	0.3349	Found associated significantly with plasma HDL-C levels in AA families. Reported to disrupt of miR-410 seed site; Reported significance for HDL-C	Shetty et al., 2015 [38] Pirim et al., 2014 [17] Evans et al., 2013 [16] Richardson et al., 2013 [39] Deo et al., 2009 [3]
rs1059611	32982 T>C	0.115	0.1276	Reported significance (p = 0.007) with TG Reported significance (p = 0.04) with HDL-C	Evans et al., 2013 [16] Bentley et al., 2014 [5]
rs9644636	33315T>G	0.58	0.1793	Reported significance with HDL-C	Bentley et al., 2014 [5]

Other variants found worthy of further investigation included those in the 3’UTR that were previously demonstrated to have opposing effect on TG levels [16]. These include rs13702 (also reported by Deo et al. [3] as a potential marker for HDL-C levels), rs1803924, rs1059611, rs3208305, rs3735964, rs3200218 all of which are found in the region important for miRNA binding indicating a potential role in gene expression under epigenetic mechanisms. Interestingly, one of the two novel variants (KUA LPL-45) identified in Kuwait Arabs was found within the region of rs13702, rs1059611 and rs3208305 of the 3’UTR. Evans et al. [16] reported a predicted functional role of rs13702 as being the site for miRNA-410 binding is lost. Although no significant association was observed for KUA LPL-44 and 45 in our study, preliminary findings indicate the importance of investigating SNPs in this region in association studies of dyslipidemia.

Other variants in non-coding regions that may also be considered include rs74304285 in intron 2 [18], rs281 and rs295 in intron 6, rs304 of intron 7 and rs320 [17]. These variants may affect the organization of the exons in the region (exons 3–8) that are important for the enzyme structure and activity. Variants rs326 of intron 8 and rs329 of intron 9 were also reported to be significantly associated with TG levels in NHW [17] and AA [18] and were identified as worthy of investigation. Variants shown to be affected by local ancestry [3, 5] were identified in Kuwaiti Arabs included rs328 (intron 3), rs343 and rs10283151 in intron 9 and rs9644636 near the 3’ end of the gene. These appear to have and independent effect on HDL-C levels [3]. However, two of the top model SNPs (rs2197089 and rs6651471) for HDL-C and three of the four for TG levels (rs10096633 near the 3’ end; rs1031045 and rs11995036 in intron1) were not identified in any of the samples re-sequenced (n = 100). The only SNP (rs3779788), located in intron 1, reported by Deo et al. [3] was identified in 8% of the samples re-sequenced with no specific distribution into a particular TG/HDL extreme.

Moreover, the SNPs located mainly in introns 6 and 9 (rs74304285; rs328; rs12679834 for TG and for HDL-C: rs256, rs328; rs1059611) reported by Bentley et al. [5] were also identified in Kuwaiti Arabs except for rs201109344. These SNPs were reported to have an effect on either or both TG and HDL-C levels dependant on the ethnic background of the population analysed [5]. It has also been suggested that some variants such rs328 may be associated with favourable lipid levels in specific ethnic groups [3, 5]. The short list of 32 variants provided (Table 6) can serve as potential markers in genetic association studies of larger cohorts of Arab ethnicity as a reference list for other understudied ethnic groups. The variants correlated to variation in plasma lipid levels and as such can be used to address some factors influencing lipid levels and attempt to fine tune the acceptable reference range for these parameters. Studies have demonstrated that the specific variants at the *LPL* gene locus (mainly in the non-coding regions) can have variable effects that correlates to the ancestry of the population tested [3, 5]. The finding here support that genetic factors do account for interethnic variation of plasma levels [3, 5].

## Conclusion

The strength of the present study rests with the inclusion of the whole *LPL* gene along with its flanking sequence in a bid to identify the effect of variants in an ethnic group that is not well studied. Furthermore, the study identified a novel variant (KUA LPL-27) LPL: g.18704C>A associated with HDL-C, TG and VLDL levels. This allowed the identification of variants that maybe absent or very rare in other populations yet could be a significant contributor to plasma lipid levels. The variants identified and their description in details, which can be used for meta-analysis or for comparison of the Arab ethnicity with other ethnic groups, is summarized (Table D in S2 File). In addition, the comparative analysis allowed formulation of a list of potential variants (Table 6 & Table G in S2 File) that may serve as a guide for selection in genetic association studies of dyslipidemia in relation to different ethnic groups. The study identified a novel SNP in a noncoding region and demonstrated its opposing effect on plasma HDL-C and TG levels and proposed a mode of action for this effect. However, a limitation was the lack of LPL protein levels in the cohort. This would have been highly informative with regards to the effect of KUA LPL-27: g.18704C>A on *LPL* expression. It is strongly recommended that KUA LPL-27: g.18704C>A be investigated in other ethnic groups as well as to investigate its potential association with clinical manifestations of dyslipidemia such as diabetes mellitus and/or heart disease. In addition, the two novel insertions (LPL: c.249+606dupA in intron and KUA LPL-36InsTTT in intron 7) identified in all the 100 samples of Kuwaiti Arabs re-sequenced presents an opportunity for further analysis in a large cohort to assess their effect on LPL activity or determine their role in the population structure of Arab ethnicity.

## Supporting information

S1 File Fig A in S1 File. Cluster analysis of the sample distribution based on TG (a) and HDLC (b) levels based on age and sex (c, d) for the 100 Kuwaiti Arab samples sequenced at the *LPL* gene locus as well as the combined TG and HDL-C distribution (e). Fig B in S1 File. Distribution of all the variants (SNPS & InDels) identified across the *LPL* gene locus in the Kuwaiti Arab samples re-sequenced (n = 100). Fig C in S1 File. Distribution of the identified SNPs at the LPL gene locus in Kuwaiti Arabs based on their minor allele frequency distribution (MAF). Fig D in S1 File. Distribution of the differences in allelic frequencies (±0.075) between the two extreme phenotypes for the 222 identified by resequencing the full LPL gene locus in 100 samples of Kuwaiti Arabs and previously reported SNPs at the extreme levels of (a) high and low triglycerides (HTG-LTG) and of (b) high and low HDL (HHDL-LHDL). Fig E in S1 File. Distribution of the differences in allelic frequencies between the two extreme phenotypes for the 47 novel variants identified by resequencing the full LPL gene locus in 100 samples of Kuwaiti Arabs at the both extreme levels of high and low triglycerides (HTG-LTG) and of high and low HDL (HHDL-LHDL). The arrows indicate those selected for validation and those with a star failed validation by Real-Time PCR. The stars indicate the variants selected for validation yet failed synthesis by real-time PCR. Fig F in S1 File. A sample of Real-Time PCR allelic discrimination plots with allele X on the x-axis against allele Y on the y-axis. The plot shows three clusters, and near the origin, the no Template Control (NTC) (n = 1). This figure illustrates the assay for the genotyping the novel variants KUA-LPL 27(a) and KUA-LPL 28 (b) These clusters are for the wildtype allele homozygote cluster represented by the blue dots, mutant allele homozygote cluster represented by the red dots and the green dots represent the heterozygote cluster. The points in each cluster are grouped closely together, and each cluster is well separated from the other clusters.
(PDF)

S2 File Table A in S2 File. **A summary of the (A) 74 designed primers (Primer 3 software) and their sequennce (B) used to amplify the target sequence of the full *LPL* gene locus in 100 Kuwaiti Arab samples**. Table B in in S2 File. **General PCR conditions used for the amplification of the *LPL* 74 overlapping target regions**. Table C in S2 File. **The volumes and final concentrations used for the amplification of the *LPL* 74 overlapping target regions**. Table D in S2 File. **A summary of all 293 variants identified by re-sequencing the *LPL* gene locus with the 74 newly designed primer sets in 100 Kuwaiti Arab samples**. The number of variants identified by gene location is shown. Table E in S2 File. **A summary of the genotypic distribution, based on the minor allele frequency, for all the identified variants (n = 293) among the five groups analyzed (n = 100).** Table G in S2 File. **List of 46 potential SNPs at the *LPL* gene locus for genetic association studies with their reported frequencies in this study and other selected studies.** Table H in S2 File. **Analysis of the distribution of variants in the introns at the *LPL* gene locus.**
(PDF)

S3 File Table F in S3 File. A summary of all the variants identified and reported at the *LPL* gene locus. The rs number, genomic position, MAF and predicted effect based on *LPL* variations assembly based on GRch38.p10 is provided.
(XLSX)
