# Supplementary material for: A novel LPL intronic variant: g.18704C>A identified by re-sequencing Kuwaiti Arab samples is associated with high-density lipoprotein, very low-density lipoprotein and triglyceride lipid levels
Source: PLoS One. 2018 Feb 13;13(2):e0192617. doi: 10.1371/journal.pone.0192617 (PMC5811003; doi:10.1371/journal.pone.0192617)
Supplement: S1 File — Fig A in S1 File. Cluster analysis of the sample distribution based on TG (a) and HDLC (b) levels based on age and sex (c, d) for the 100 Kuwaiti Arab samples sequenced at the LPL gene locus as well as the combined TG and HDL-C distribution (e). Fig B in S1 File. Distribution of all the variants (SNPS & InDels) identified across the LPL gene locus in the Kuwaiti Arab samples re-sequenced (n = 100). Fig C in S1 File. Distribution of the identified SNPs at the LPL gene locus in Kuwaiti Arabs based on their minor allele frequency distribution (MAF). Fig D in S1 File. Distribution of the differences in allelic frequencies (±0.075) between the two extreme phenotypes for the 222 identified by resequencing the full LPL gene locus in 100 samples of Kuwaiti Arabs and previously reported SNPs at the extreme levels of (a) high and low triglycerides (HTG-LTG) and of (b) high and low HDL (HHDL-LHDL). Fig E in S1 File. Distribution of the differences in allelic frequencies between the two extreme phenotypes for the 47 novel variants identified by resequencing the full LPL gene locus in 100 samples of Kuwaiti Arabs at the both extreme levels of high and low triglycerides (HTG-LTG) and of high and low HDL (HHDL-LHDL). The arrows indicate those selected for validation and those with a star failed validation by Real-Time PCR. The stars indicate the variants selected for validation yet failed synthesis by real-time PCR. Fig F in S1 File. A sample of Real-Time PCR allelic discrimination plots with allele X on the x-axis against allele Y on the y-axis. The plot shows three clusters, and near the origin, the no Template Control (NTC) (n = 1). This figure illustrates the assay for the genotyping the novel variants KUA-LPL 27(a) and KUA-LPL 28 (b) These clusters are for the wildtype allele homozygote cluster represented by the blue dots, mutant allele homozygote cluster represented by the red dots and the green dots represent the heterozygote cluster. The points in each cluster are grouped [file pone.0192617.s001.pdf]

Supplementary Information

S1: Figures A-E

A novel *LPL* intronic variant: g.18704C>A identified by re-sequencing Kuwaiti Arab samples is associated with high-density lipoprotein, very low-density lipoprotein and triglyceride lipid levels

Suzanne A. Al-Bustan [1*]; Ahmad Al-Serri[2]; Babitha G. Annice[1]; Majed A. Alnaqeeb[1]; Wafa Y. Al-Kandari[1]; Mohammed Dashti[3]

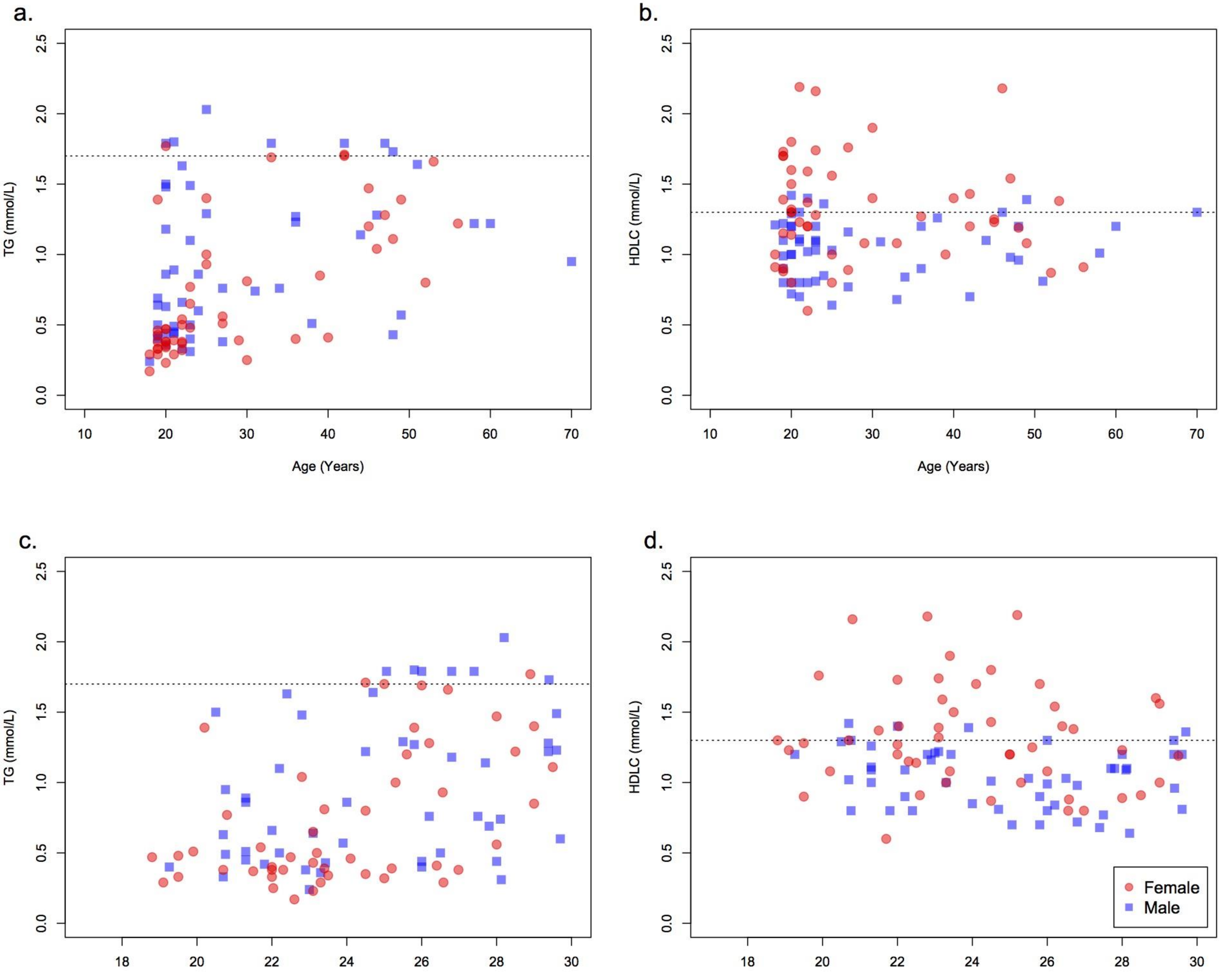

a.
TG (mmol/L)
Age (Years)
b.
HDLC (mmol/L)
Age (Years)
c.
TG (mmol/L)
BMI
d.
HDLC (mmol/L)
BMI
Female
Male

e.

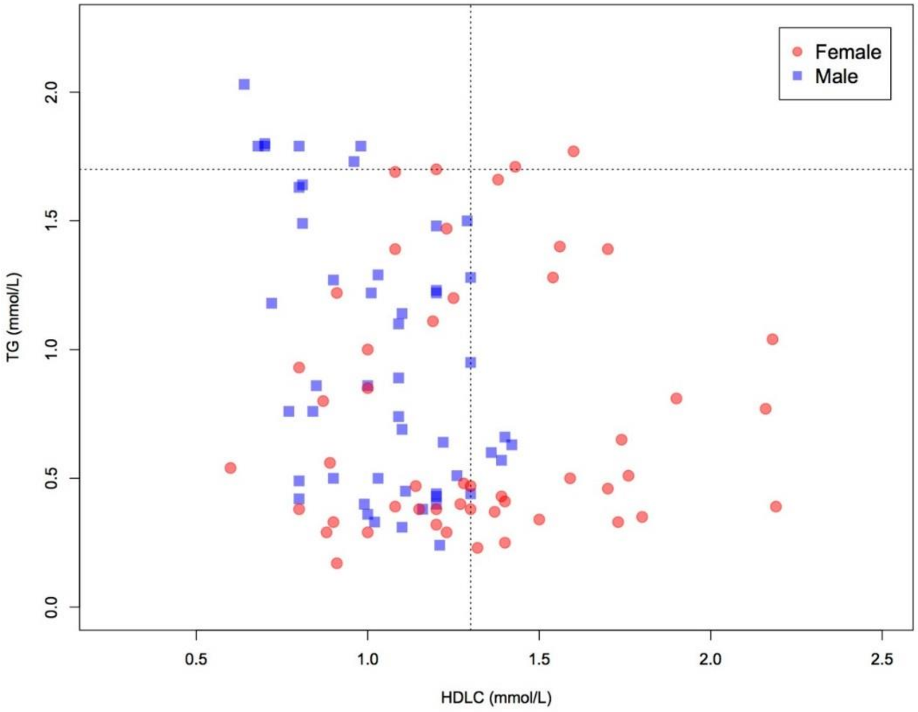

Figure A. Cluster analysis of the sample distribution based on TG (a) and HDLC (b) levels based on age and sex (c, d) for the 100 Kuwaiti Arab samples sequenced at the *LPL* gene loci as well as the combined TG and HDL-C distribution (e). The vertical and horizontal dotted lines represent the cut-off range.

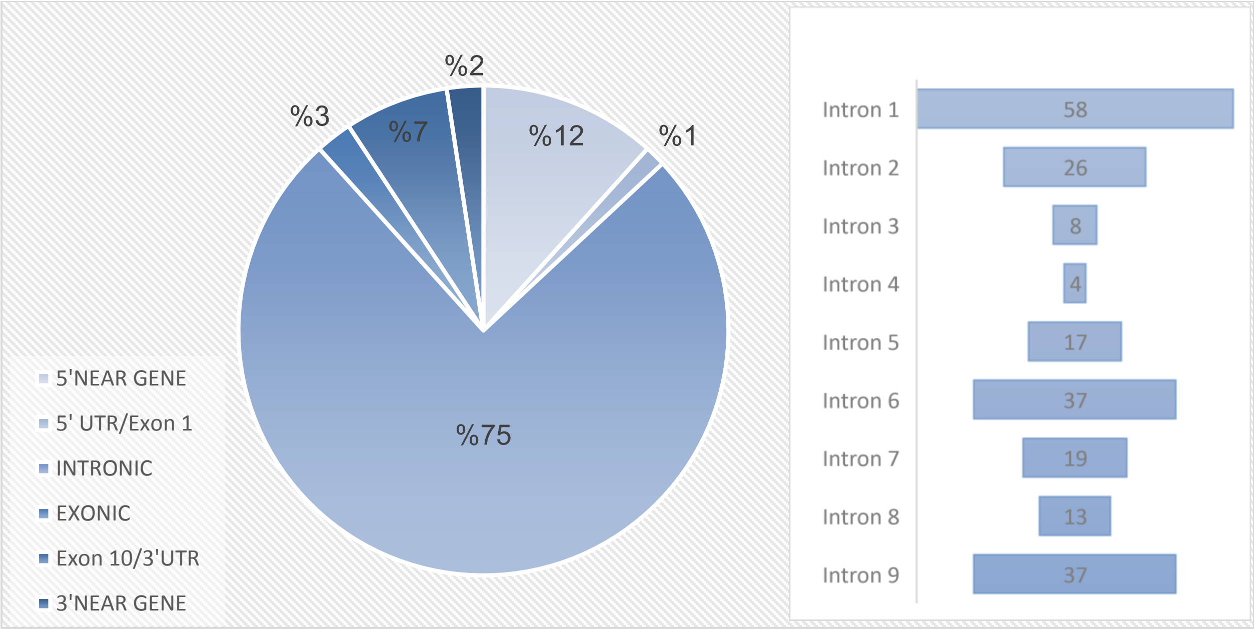

Figure B. Distribution of all the variants (SNPS & InDels) identified across the LPL gene locus in the Kuwaiti Arab samples re-sequenced (n=100).

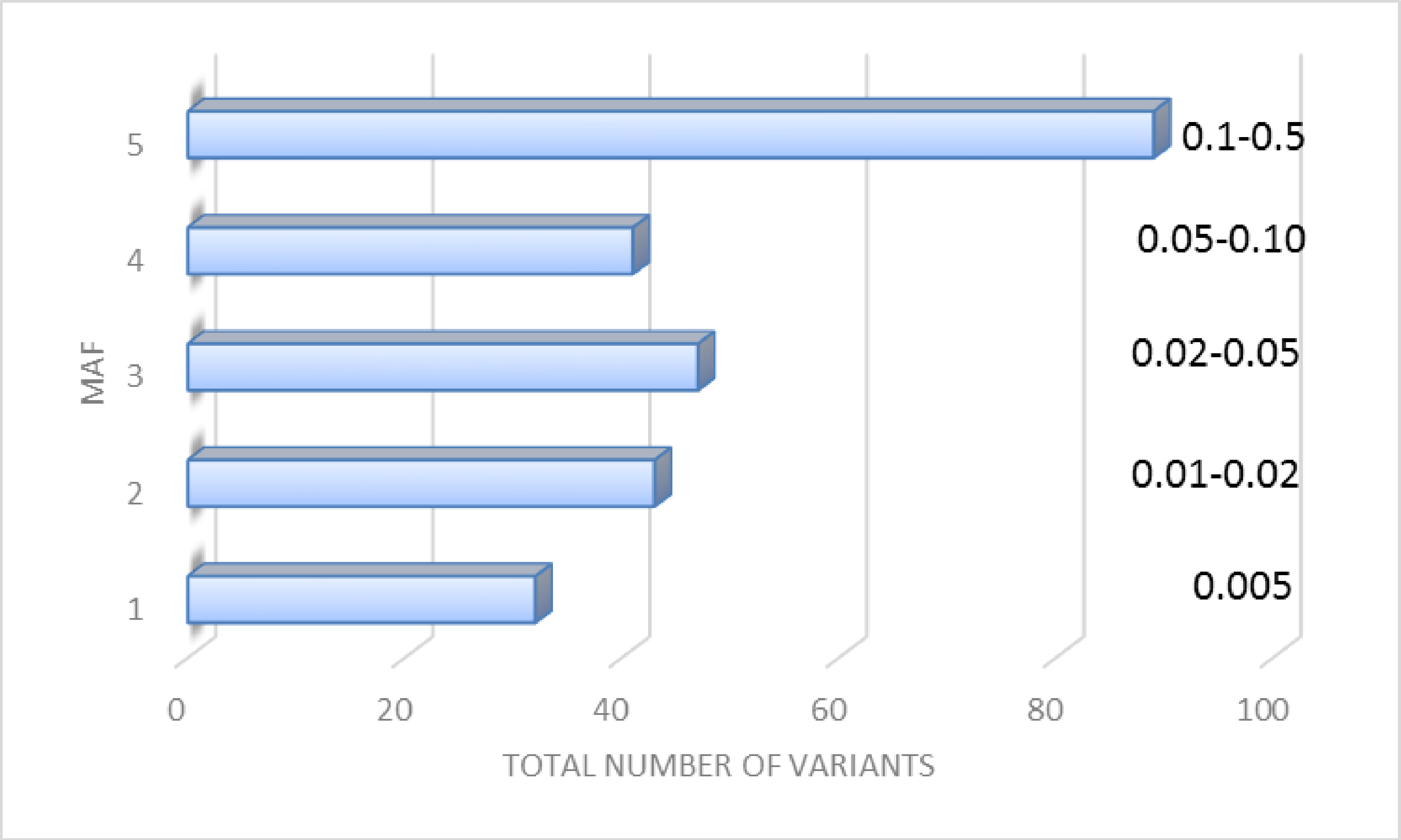

Figure C. Distribution of the identified SNPs at the *LPL* gene locus in Kuwaiti Arabs based on their minor allele frequency distribution (MAF).

a.

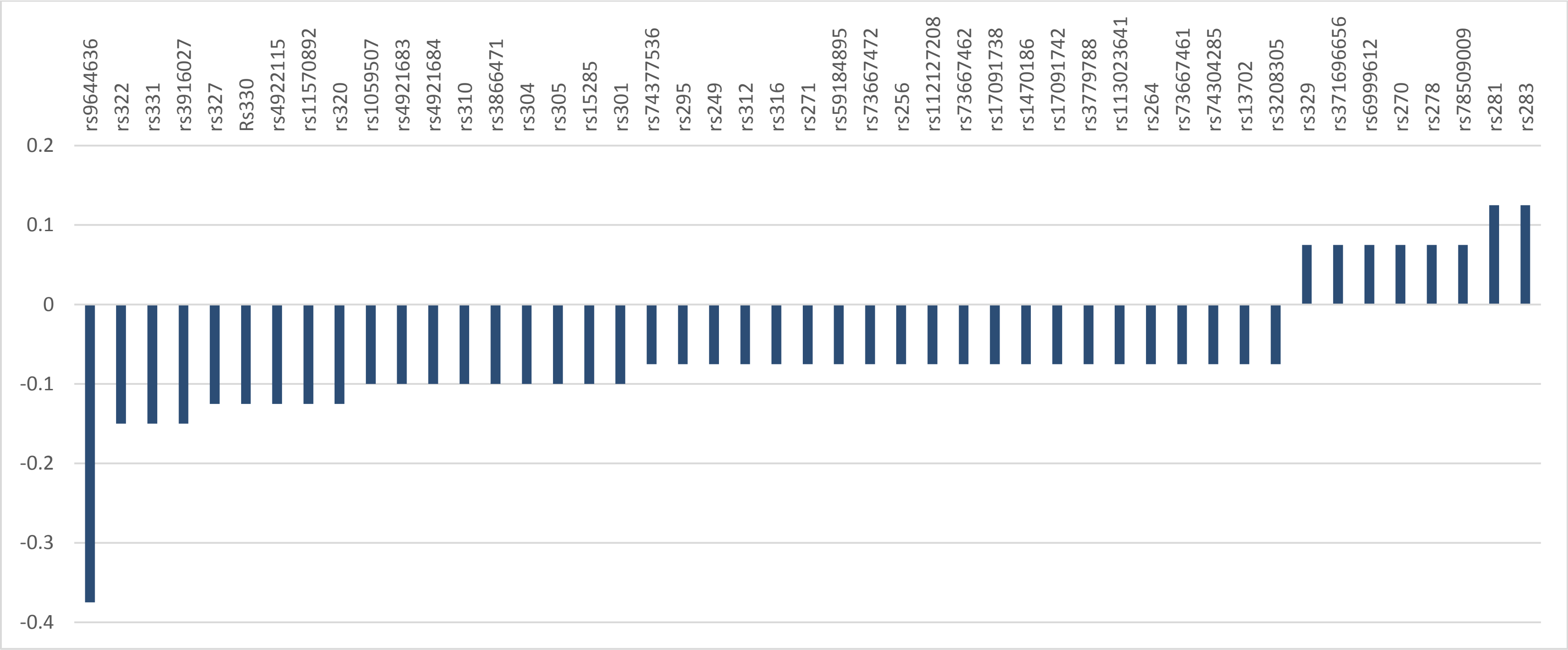
rs9644636
rs322
rs331
rs3916027
rs327
Rs330
rs4922115
rs11570892
rs320
rs1059507
rs4921683
rs4921684
rs310
rs3866471
rs304
rs305
rs15285
rs301
rs74377536
rs295
rs249
rs312
rs316
rs271
rs59184895
rs73667472
rs256
rs112127208
rs73667462
rs17091738
rs1470186
rs17091742
rs3779788
rs113023641
rs264
rs73667461
rs74304285
rs13702
rs3208305
rs329
rs371696656
rs6999612
rs270
rs278
rs78509009
rs281
rs283
0.2
0.1
0
-0.1
-0.2
-0.3
-0.4

b.

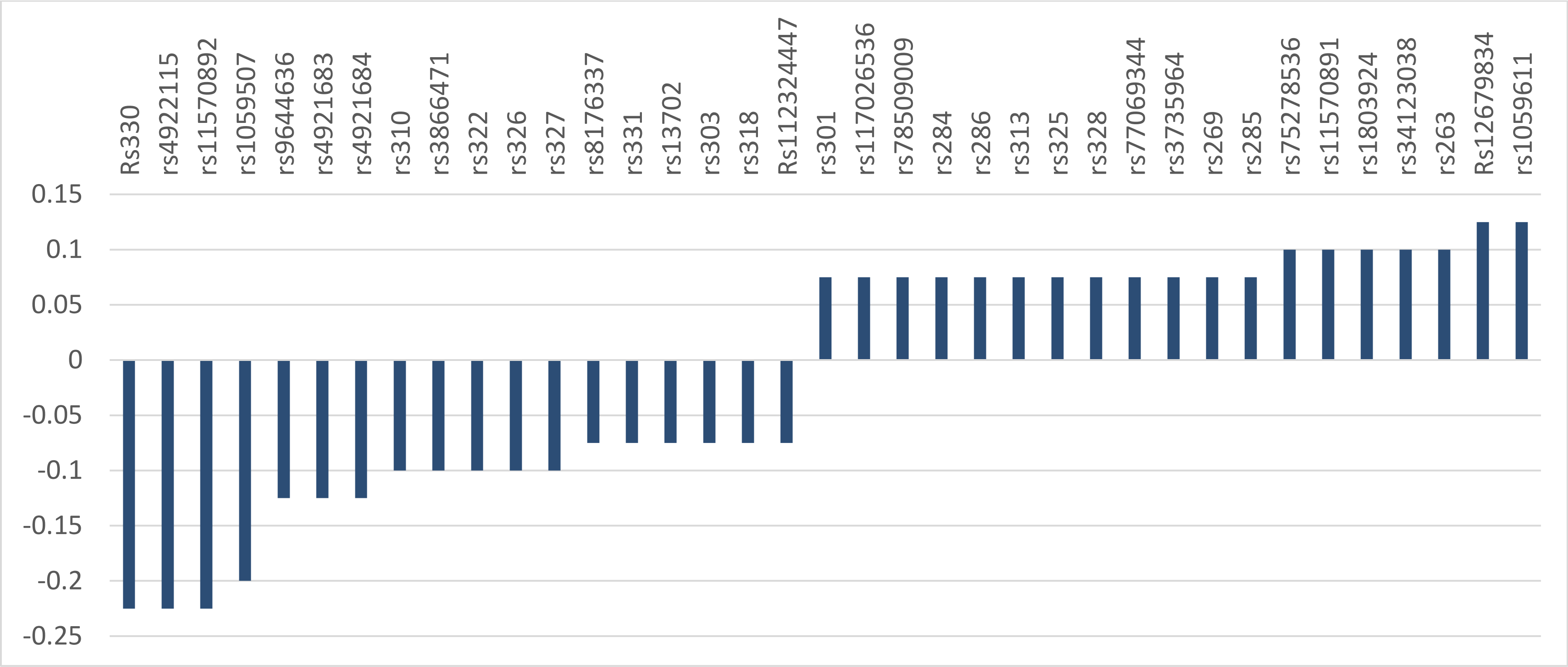

Figure D. Distribution of the differences in allelic frequencies (±0.075) between the two extreme phenotypes analysed for the 222 identified by resequencing the full *LPL* gene locus in 100 samples of Kuwaiti Arabs and previously reported SNPs at the extreme levels of (a) high and low triglycerides (HTG-LTG) and of (b) high and low HDL (HHDL-LHDL).

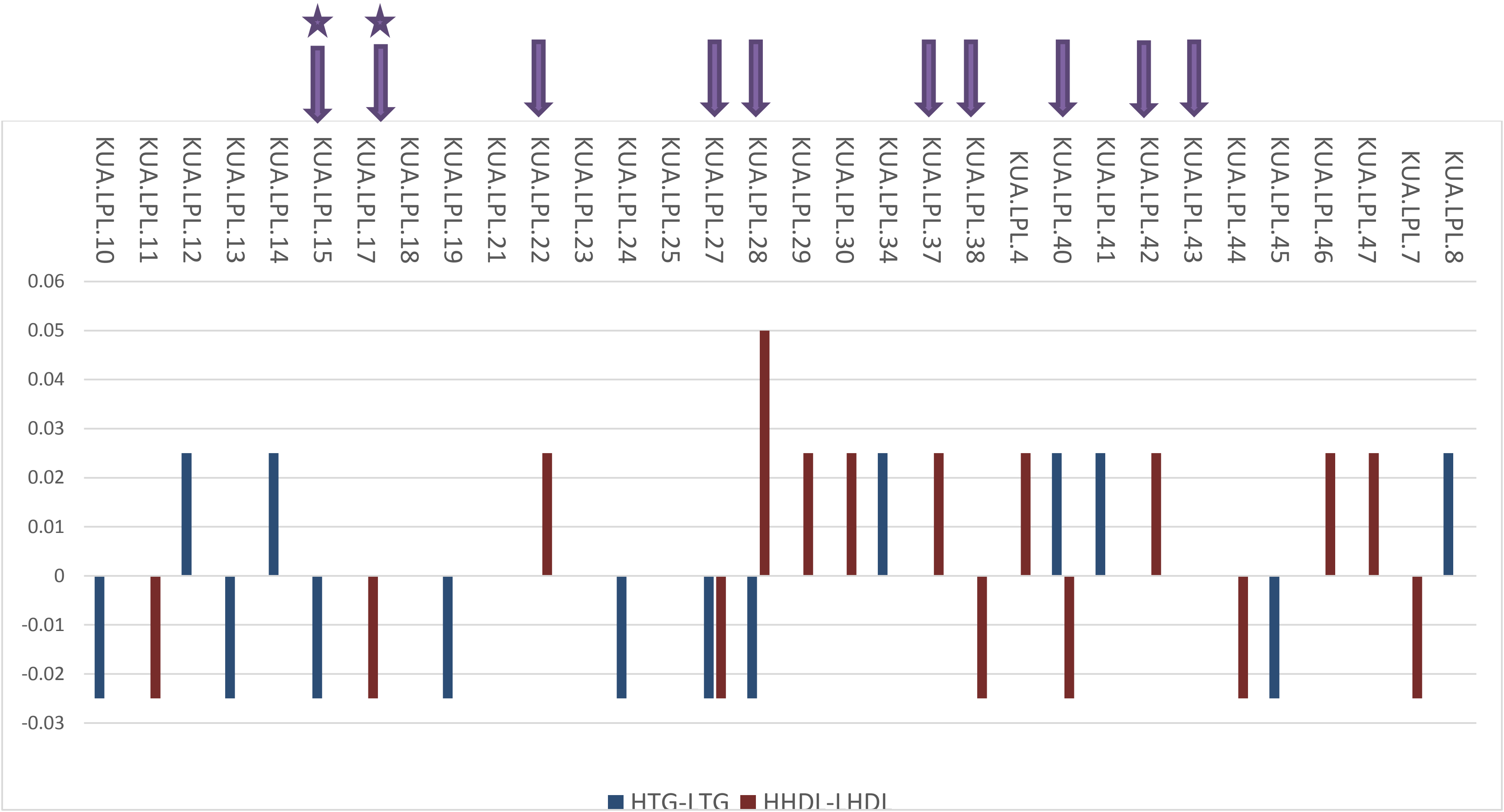

Figure E. Distribution of the differences in allelic frequencies between the two extreme phenotypes analysed for the 47 novel variants identified by resequencing the full *LPL* gene locus in 100 samples of Kuwaiti Arabs at the both extreme levels of high and low triglycerides (HTG-LTG) and of high and low HDL (HHDL-LHDL). The arrows indicate those selected for validation and those with a star failed validation by Real-Time PCR. The stars indicate the variants selected for validation yet failed synthesis by real-time PCR.

a. b.

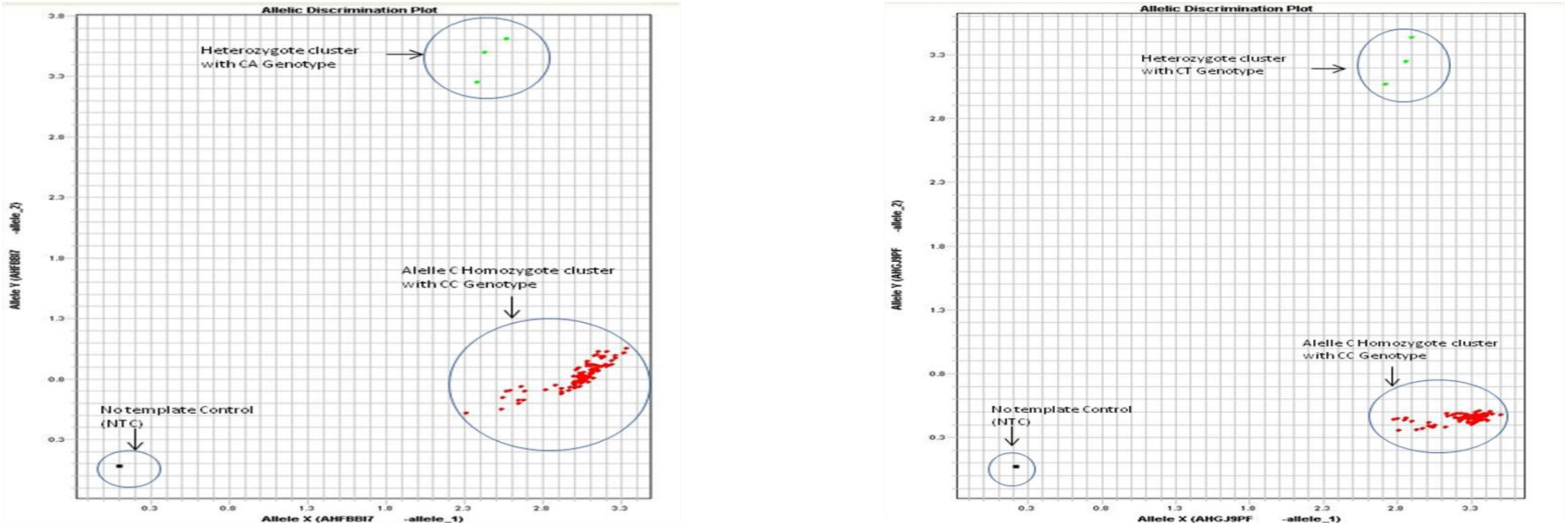

Figure F. A sample of Real-Time PCR allelic discrimination plots with allele X on the x-axis against allele Y on the y-axis. The plot shows three clusters, and near the origin, the no Template Control (NTC) (n=1). This figure illustrates the assay for the genotyping the novel variants KUA-LPL 27(a) and KUA-LPL 28 (b) These clusters are for the wildtype allele homozygote cluster represented by the blue dots, mutant allele homozygote cluster represented by the red dots and the green dots represent the heterozygote cluster. The points in each cluster are grouped closely together, and each cluster is well separated from the other clusters.
