# Supplementary material for: A novel LPL intronic variant: g.18704C>A identified by re-sequencing Kuwaiti Arab samples is associated with high-density lipoprotein, very low-density lipoprotein and triglyceride lipid levels
Source: PLoS One. 2018 Feb 13;13(2):e0192617. doi: 10.1371/journal.pone.0192617 (PMC5811003; doi:10.1371/journal.pone.0192617)
Supplement: S2 File — Table A in S2 File. A summary of the (A) 74 designed primers (Primer 3 software) and their sequennce (B) used to amplify the target sequence of the full LPL gene locus in 100 Kuwaiti Arab samples. Table B in in S2 File. General PCR conditions used for the amplification of the LPL 74 overlapping target regions. Table C in S2 File. The volumes and final concentrations used for the amplification of the LPL 74 overlapping target regions. Table D in S2 File. A summary of all 293 variants identified by re-sequencing the LPL gene locus with the 74 newly designed primer sets in 100 Kuwaiti Arab samples. The number of variants identified by gene location is shown. Table E in S2 File. A summary of the genotypic distribution, based on the minor allele frequency, for all the identified variants (n = 293) among the five groups analyzed (n = 100). Table G in S2 File. List of 46 potential SNPs at the LPL gene locus for genetic association studies with their reported frequencies in this study and other selected studies. Table H in S2 File. Analysis of the distribution of variants in the introns at the LPL gene locus. (PDF) [file pone.0192617.s002.pdf]

Supplementary Information

S2: Tables A-E and G-H

A novel *LPL* intronic variant: g.18704C>A identified by re-sequencing Kuwaiti Arab samples is associated with high-density lipoprotein, very low-density lipoprotein and triglyceride lipid levels

Suzanne A. Al-Bustan [1*]; Ahmad Al-Serri[2]; Babitha G. Annice[1]; Majed A. Alnaqeeb[1]; Wafa Y. Al-Kandari[1]; Mohammed Dashti[3]

Supplementary Tables

Table A. A summary of the (i) 76 designed primers (Primer 3 software) and their sequennce (ii) used to amplify the target sequence of the full *LPL* gene locus in 100 Kuwaiti Arab samples.

i.

Primer Set	LPL region	Target Size	Primer Set	LPL region	Target Size	Primer Set	LPL region	Target Size
1	5' NEAR GENE	500	26	Intron 1	628	51	Intron 6	403
2		486	27		574	52		698
3		549	28		590	53		685
4		682	29		586	54		571
5		625	30		606	55	Intron 7	695
6		559	31	Exon 2	563	56		550
7		498	32	Intron 2	499	57		502
8		571	33		654	58		677
9		569	34		588	59	Exon 8	637
10		647	35		675	60	Intron 8	700
11		656	36		602	61		698
12		680	37		640	62	Exon 9	508
13	5' UTR	447	38		529	63	Intron 9	525
14	Exon 1	688	39		523	64		648
15	Intron 1	626	40	Exon 3	553	65		424
16		622	41	Intron 3	601	66		613
17		688	42		667	67		631
18		588	43	Exon 4	557	68		465
19		568	44	Intron 4	644	69		632
20		659	45	Intron 5	650	70	3’ UTR	665
21		673	46		603	71		667
22		609	47		576	72		506
23		551	48		456	73		667
24		650	49	Intron 6	498	74	3’ NEAR GENE	689
25		408	50		578			

* The annealing temperature was 65°C for all the primers.

ii.

	Forward Primer	Reverse Primer
1	TCCAGCAGCACATCAAAAAG	ATTTTGGGCAGAGATGATGG
2	ACTGAATGGGCAAAAACTGG	GCGTGGAATGTTCTTCCATT
3	TCCCATTCACAATTGCTTCA	CAGTTTTCCCAGCACCATTT
4	AAATGGTGCTGGGAAAACTG	CCCTGAGGAATCACCACACT
5	GCTGGAGAGGATGTGGAGAG	GGGTAAATGTGCACAACGTG
6	ACACCAACATGGCACAGGTA	AATTGCACTGGGCACTTTTC
7	TGATTCCCAGCCTATTCCTG	ACTCGGCCTTCAGTAGCTCA
8	CAGTGGCAAGTGTCCTGCTA	CTGCACTTCTGTGCCTTTCA
9	GCAGCCAATCAGGAGAGAAC	AGACCATGCAAGGGATGAAT
10	GCCATTCACACCATGAACAC	GCTTTTCTGCCAAAATGCTT
11	GCATTTTGGCAGAAAAGCAT	CTTGCAGTCCTCCCTCACTC
12	GAGTTGTGCAGCATCAGCAT	CGCTTAGCTTAAGGGGTTCC
13	GAGTGAGGGAGGACTGCAAG	AGCTTTCCCTTGAGGAGGAG
14	GGCAGGGTTGATCCTCATTA	CAGCCAGAGTGGAAGGGATA
15	CCCGAACTGAGGATGAGAAG	CTGGAAGCTGGAGAGAGTCC
16	GTGTCGGGGTGGAGAAAGTA	GGCCATCAAAGCTATGGAGA
17	CACGGGACAACATTTCCTTT	TGGAGTTGGCTCCTACCATC
18	GATGGTAGGAGCCAACTCCA	GGACAGTGCCTCTTGTCCTC
19	ATCACCTCCTCTGGGCTCTT	TCATTTTGATGGCTGGAACA
20	CGTGACCACCACAAAGACTG	GAGCACAGACTGGGAAGAGC
21	TGCCGGAAGTGAGCCTATTA	GGATGGCAAATGGAAACATC
22	AGATCCTCAACAGCCCTTCA	CCACTGTGCCAGCCTACTTT
23	AAAGTAGGCTGGCACAGTGG	CCCCCTTTTCTCTCACTCCT
24	GGGACATGGCAAAAGACAGT	CTGGAATTGCTGGTGGTTTT
25	TCATTTCAAAACCACCAGCA	TTCCCAACCAGAAAGCACTC
26	GGGCACTGAAGGCAGAATTA	TGAGGCTGCGAGGACTACTT
27	AAATGTTGGCCAATCCTCAG	TGTTCCACACAAAGGCAAAA
28	TTTTGCCTTTGTGTGGAACA	GTTCACTGCACCCTCGAACT
29	CAAAAGCCTCCTCATGCTTC	TGCTTTCGTGGAGAACACTG
30	CAGTGTTCTCCACGAAAGCA	TCACCATGAAGGTTTTGCTG
31	GTCCGTGGCTACCTGTCATT	TAAATGGAGGCCCAGAGATG
32	CATCTCTGGGCCTCCATTTA	AGCGATTGTCCTCTTCAGTCA
33	TGCTGACATGCCAGATGATT	AGAATCTCTTATGCCCAGGTG
34	CTGAAGAGGACAATCGCTACAA	TTTCCCACAACTTCCCAAAG
35	CCACCTGGGCATAAGAGATT	GTGGCTGGCTTAGTTCTTGC
36	CGGTGCCACTTCCTATCATT	CAAGCACATACCACCACACC
37	GTTGGATGTCCAGCCTTTTT	TTCGTCTGTGCCTGTGAGTC
38	CCTGGGCGAGACAGTAAGAC	CACCTGGCTAGGAGCAGAAC
39	AGGCTCTCCAGCTGTGTCAT	GACAGGTCTGTCCCTTCCAA
40	ACCCACTTTGCATTCAGACC	CCCAGTCTTACCTCCATCCA
41	TACACCAAACTGGTGGGACA	CTATGCACCTTCCCTTCCAA
42	CTAAGTGGGCCCAACAGAAA	GCCAAGCAATGACAGACTCA

43	CTGTTAAATGAGGGGCTGGA	TGGTCAGACTTCCTGCAATG
44	CTTGGGATACAGCCTTGGAG	CACTCAGGATGGCTGGATTT
45	AAATCCAGCCATCCTGAGTG	TGTCTGCCTGGCTCTAAGGT
46	GGGCATGTTGACATTTACCC	CAACATTGCAAGTCCCTGTG
47	GGGTGCAGTCACACAATCAC	CTGCCCCTTGCCTTACAATA
48	TATGATGGGTTTTGGGGGTA	AGCCCTTTCTCAAAGGCTTC
49	TCCAAGGAAGCCTTTGAGAA	GGTGGATGGTTCCAAATGAG
50	ACTAAACCCTGAGCCCTGGT	GAATGTGTGTGGCTGTGTCC
51	GGACACAGCCACACACATTC	TCCGAAGACCTCCTCTCTCA
52	TCAAGCTTGGCCTCTTGTTT	CCCTAGGGGACACTGACAGA
53	ATGGCACCCATGTGTAAGGT	TGAGCGAGACTGTGTCTCAAA
54	TTTTAGCAGCTGTGGGGTTT	GAACCATGATGATGGAGGAAA
55	TGGCCTCCAAGAACTCTTTT	CCTTAGCATCTGGAGCAAGC
56	GCTTGCTCCAGATGCTAAGG	TGGCCAATTTGAAGCATTTT
57	CTGAGCAGCAGAAGCAGAGA	CATGGTGAGACCCTGTCG
58	GCAATGGTAACAGGAAACCAC	CACCTTGTCTGCACTGGCTA
59	ATGCCCAGCCTACCCTTTAC	AATGAAGGTCGATGGCATTT
60	GGCAGTGGGTAAATGAATCG	GATCTTCTGAATGGCGAAGC
61	AGGCCTGAAGTTTCCACAAA	AGTTTGCAAAATCCCAGCAC
62	GCCTTCACAATTCAGGGAGA	GGGCTTTCACCAAGAGATGA
63	AGGCAGATGCCCTAATTCCT	CATGAAGCTGCCTCCCTTAG
64	GCTAAAGCTGACTGGGCATC	GCAGCTTTGAGGGTTTTGAG
65	CTGCCAAGCAAACAGAATGA	TATAATGGCGCGATCTCATC
66	ATCGCGCCATTATATTCCAG	TTGCCTGGCTCAATACCACT
67	GTGGTATTGAGCCAGGCAAA	CGACTGGACGTGCTTTACAA
68	CGGCCCTAGATGCAGTTTTA	TGAGACAGGCACATCACCTG
69	TGCTTGTAAAGCACGTCAG	GAAAGGCCTGTTCCATTTCA
70	TCAGGTGATGTGCCTGTCTC	TGGAAAAAGCCTTCAAATGG
71	CCTCCAAAAATGATGCACCT	CCGAAAGATCCAGAATTCCA
72	GCTCCTTCATGTGGCGTATT	TCATGTCAAAGCCAGGAACA
73	AATCCCTCTCTCCCCCTTCT	TTCCCTTCCGAATTATGCTG
74	TATAGCTGGGAACCCGACTG	GCCACAATGACCTTTCCAAT
75	GTGACCCAGGGTGCATTAAC	AAATGGCCAAAGATGCCTTA
76	TGGTCTCACAGAGCCAACTC	TGCTCTTCAGCCTGGAAGTT

Table B. General PCR conditions used for the amplification of the *LPL* 74 overlapping target regions.

		35 cycles				
Steps	Initial Denaturation	Denaturation	Annealing	Extension	Final Extension	Hold at
Temperature	95^{0} C	95^{0} C	65^{0} C	72^{0} C	72^{0} C	4^{0} C
Time	5 mins	30 secs	30 secs	30 secs	7 mins	-

Table C. The volumes and final concentrations used for the amplification of the *LPL* 74 overlapping target regions.

Components	Volume	Final Concentration
Gene Amp Fast PCR Master Mix (2 X)	10ul	1 X
Double Distilled Water	2.5ul	-
Forward Primer (5um)	1.25ul	0.26 um
Reverse Primer (5um)	1.25ul	0.26um
Total Reaction Mixture	15ul	
Diluted DNA (5ng)	5ul	1.25ng

Table D. A summary of all 293 variants s identified by re-sequencing the *LPL* gene locus with the 74 newly designed primer sets in 100 Kuwaiti Arab samples. The number of variants identified by gene location is shown and is based on gene assembly GRch38.p10.

Sl. no.	Regions	Primer Sets	Nucleotide Positions	Total Variants	InDels	SNPs	Novel SNPs
1	5' NEAR GENE	P1	42-541	1	1	0	1
2	5' NEAR GENE	P2	316-801	1	1	0	1
3	5' NEAR GENE	P3A	660-1208	1	1	0	1
4	5' NEAR GENE	P4	1189-1870	1	0	1	1
5	5' NEAR GENE	P5	1772-2396	1	1	0	1
6	5' NEAR GENE	P6A	2338-2896	2	1	1	1
7	5' NEAR GENE	P7	2659-3156	7	0	7	2
8	5' NEAR GENE	P8	3038-3608	3	1	2	2
9	5' NEAR GENE	P9	3264-3832	7	0	7	1
10	5' NEAR GENE	P10A	3708-4354	3	1	2	1
11	5' NEAR GENE	P11	4337-4992	5	0	5	1
12	5' NEAR GENE	P12	4496-5175	1	0	1	1
13	5' NEAR GENE	P13	4973-5421	2	0	2	1
14	5' UTR	P14A	5231-5920	3	0	3	0
15	Exon 1	P14A	5182-5458	1	1	0	1
16	Intron 1	P15	5712-6264	2	0	2	1
17	Intron 1	P16	6245-6866	1	0	1	0
18	Intron 1	P17A	6736-7423	2	1	1	0
19	Intron 1	P18	7182-7613	1	0	1	0
20	Intron 1	P19	7908-8475	5	1	4	3
21	Intron 1	P20	8416-9074	5	0	5	1
22	Intron 1	P21A	9011-9683	2	0	2	0
23	Intron 1	P22A	9437-10045	4	0	4	0
24	Intron 1	P23	10026-10576	1	0	1	0

25	Intron 1	P24	11188-11837	7	0	7	1
26	Intron 1	P25A	11810-12217	1	0	1	0
27	Intron 1	P26	12126-12753	5	1	4	1
28	Intron 1	P27	12557-13130	4	0	4	0
29	Intron 1	P28	13111-13700	5	0	5	0
30	Intron 1	P29	13278-13863	8	2	6	1
31	Intron 1	P30	13844-14449	4	0	4	1
32	Exon 2	P30	14019-14270	1	0	1	0
33	Intron 2	P31A	14400-14962	3	0	3	0
34	Intron 2	P32	14943-15441	5	2	3	1
35	Intron 2	P34	15424-16077	2	0	2	0
36	Intron 2	P35	15693-16280	3	1	2	0
37	Intron 2	P36	16130-16804	7	0	7	0
38	Intron 2	P37	16597-17198	2	0	2	0
39	Intron 2	P38	16899-17538	1	0	1	0
40	Intron 2	P39	17210-17738	3	0	3	0
41	Exon 3	P40	17560-18082	1	0	1	0
42	Intron 3	P41	18021-18573	2	0	2	0
43	Intron 3	P42	18328-18928	3	0	3	1
44	Intron 3	P43	18854-19520	3	0	3	0
45	Exon 4	P43	19239-19351	1	0	1	0
46	Intron 4	P44A	19468-20024	4	2	2	0
47	Exon 5	P44A	20049-20283	0	0	0	0
48	Intron 5	P45	20005-20648	3	0	3	0
49	Intron 5	P46	20383-21032	4	0	4	0
50	Intron 5	P47	20996-21598	4	0	4	0
51	Intron 5	P48	21515-22090	6	0	6	2
52	Exon 6	P48	21770-22013	0	0	0	0
53	Intron 6	P49	22065-22520	5	0	5	0

54	Intron 6	P50A	22317-22814	4	2	2	1
55	Intron 6	P51	22795-23372	5	0	5	0
56	Intron 6	P52A	23303-23705	5	0	5	0
57	Intron 6	P53	23481-24178	9	4	5	2
58	Intron 6	P54	23795-24479	1	0	1	0
59	Intron 6	P55A	24440-25010	7	2	5	1
60	Intron 6	P56A	24991-25685	2	0	2	0
61	Exon 7	P56A	25189-25310	0	0	0	0
62	Intron 7	P57	25565-26114	7	2	5	3
63	Intron 7	P58	25811-26312	8	1	7	1
64	Intron 7	P59	26202-26878	3	0	3	0
65	Intron 7	P60	26541-27177	1	0	1	0
66	Exon 8	P60	26830-27013	2	0	2	0
67	Intron 8	P61	27022-27721	7	2	5	0
68	Intron 8	P62	27281-27978	2	1	1	0
69	Intron 8	P63	27901-28408	6	0	6	0
70	Exon 9	P64A	28356-28880	3	0	3	1
71	Intron 9	P65A	28877-29524	9	0	9	0
72	Intron 9	P66A	29511-29934	7	2	5	1
73	Intron 9	P67	29916-30528	9	1	8	1
74	Intron 9	P69	30506-31136	2	0	2	1
75	Intron 9	P70	30885-31349	4	1	3	2
76	Intron 9	P71A	31193-31824	6	1	5	0
77	3'UTR	P72	31745-32409	6	1	5	1
78	3'UTR	P73A	32132-32798	4	0	4	0
79	3'UTR	P74A	32703-33208	4	0	4	1
80	3'UTR	P75A	33026-33692	4	1	3	0
81	3' NEAR GENE	P76	33260-33948	7	0	7	2
	Total SNPs	-		293	39	254	47

S: subsitution; I: insertion; D: deletion

Table E. A summary of the genotypic distribution, based on the minor allele frequency, for all the identified variants (n=293) among the five groups analyzed (n=100).

Category	Variants (n)	InDels (n)	SNPs (n)	Normal	<TG	> TG	> HDL-C	< HDL-C
Ancestral "Common"	118	27	91	75.11%	79.73%	79.73%	73.52%	74.89%
Heterozygote "Common"				19.07%	18.13%	14.78%	22.42%	20.05%
Mutant "Common"				5.82%	**2.14%**	5.49%	4.07%	5.05%
Ancestral "Rare"	57	6	51	92.74%	95.20%	94.31%	94.22%	95.20%
Heterozygote "Rare"				6.96%	4.8%	5.49%	5.78%	4.80%
Mutant "Rare"				0.10%	0.0%	0.20%	0.0%	0.0%
Ancestral "Very Rare"	118	6	112	99.06%	99.02%	98.39%	98.57%	98.93%
Heterozygote "Very Rare"				0.94%	0.94%	1.61%	1.43%	1.07%
Mutant "Very Rare"				0.0%	0.04%	0.0%	0.0%	0.0%
Ancestral "Novel"	47	15	32	97.97%	99.06%	98.75%	98.28%	98.75%
Heterozygote "Novel"				2.03%	0.94%	1.25%	1.72%	1.25%
Mutant "Novel"				0.0%	0.0%	0.0%	0.0%	0.0%

Table G. List of 46 potential SNPs at the *LPL* gene locus for genetic association studies with their reported frequencies in this study and other selected studies.

SNP	Position	NLP (N=20)	HTG (n=20)	LTG (n=20)	KUW TGΔF (n=40)	H HDL (n=20)	L HDL (n=20)	KUW HDL-CΔF (n=50)	KMAF (n=100)	GMAF	Pirim et al., 2015 (AA)	Pirim et al., 2014 (NHW)	Evans et al., 2013 (HTG; LTG)
	**Exon 2**												
rs1801177	14127G>A	0.05	0	0	0	0	0	0	0.01	0.0176	0.05		
	**Intron 2**												
**rs34123038**	15574G>A	0.1	0.05	0.025	-0.025	0.125	0.025	0.1	0.065	0.0212			
**rs74304285**	16449G>A	0.05	0.125	0.05	-0.075	0.125	0.075	0.05	0.085	0.1396	0.056		
rs8176337	15090C>G	0.25	0.225	0.2	-0.025	0.25	0.325	-0.075	0.25	0.346	0.313	0.242	
	**Exon 4**												
**rs248**	19245G>A	0.5	0	0.025	0.025	0.025	0.075	-0.05	0.035	0.0387		0.067	
	**Intron 4**												
rs252	19651delA				NA			NA	0.5	0.4543	0.093		
	**Intron 5**												
rs263	21231 C>T	0.225	0.2	0.2	0	0.25	0.15	0.1	0.205	0.2476			
	**Intron 6**												
**rs281**	23442A>T	0.225	0.125	0.25	0.125	0.100	0.150	-0.050	0.17	0.3129			
**rs283**	23517C>T	0.2	0.075	0.2	0.125	0.100	0.100	0.000	0.135	0.264			
**rs295**	24657 A>C	0.25	0.2	0.125	-0.075	0.3	0.275	0.025	0.23	0.2746	0.39	0.223	
rs294	24544 T>C	0.125	0.125	0.1	-0.025	0.125	0.125	0	0.12	0.1322		0.118	
rs282	23445C>G	0.15	0.05	0.05	0	0.125	0.1	0.025	0.095	0.0585			
rs277	22822T>C	0.075	0.5	0.075	0.025	0.025	0.025	0	0.05	0.1378		0.196	
**rs279**	23115C>G	0.025	0.025	0	-0.025	0.05	0	0.05	0.02	0.0387	0.15		
rs286	23675 A>T	0.5	0.025	0.05	0.025	0.125	0.05	0.075	0.06	0.0331		0.095	
	**Intron 7**												
**rs301**	25353T>C	0.25	0.225	0.125	-0.1	0.3	0.225	0.075	0.225	0.382	0.288		
**rs304**	25780T>G	0.25	0.225	0.125	-0.1	0.3	0.275	0.025	0.235	0.2532			
**rs305**	25820A>G	0.25	0.225	0.125	-0.1	0.3	0.275	0.025	0.235	0.2526			
rs310	25965C>T	0.125	0.1	0	-0.1	0.075	0.175	-0.1	0.095	0.1066			
rs327	27955T>G	0.275	0.275	0.15	-0.125	0.325	0.425	-0.1	0.29	0.2925		0.265	
	**Intron 8**												
rs326	27858A>G	0.325	0.3	0.375	-0.05	0.375	0.475	-0.1	0.345	0.3494			
rs316	26855C>A	0.15	0.15	0.075	-0.075	0.175	0.2	-0.025	0.15	0.1526	0.225	0.113	
**rs320**	32067 A>T	0.15	0.15	0.025	-0.125	0.375	0.075	0.05	0.335	0.3375	0.306	0.258	
	**Exon 9**												
rs328	28143C>G	0.1	0.05	0.05	0	0.125	0.05	0.075	0.075	0.0925		0.103	
	**Intron 9**												
**rs329**	28505A>G	0.05	0	0.075	0.075	0.025	0.05	-0.025	0.04	0.0369	0.097		

rs330	28815G>A	0.125	0.2	0.075	-0.125	0.1	0.325	-0.225	0.175	0.1244	0.085		
Rs12679834	28852T>C	0.1	0.075	0.05	0.025	0.175	0.05	0.125	0.09	0.0972	0.058		
rs75278536	29844T>G	0.1	0.05	0.05	0	0.15	0.05	0.1	0.08	0.0935			
rs11570891	31229C>T	0.1	0.05	0.05	0	0.175	0.075	0.1	0.09	0.0931		0.107	
	**3'UTR**												
rs4922115	31249 G>A	0.175	0.225	0.1	-0.125	0.075	0.3	-0.225	0.175	0.1254		0.146	0.056; 0.138
rs11570892	32036 A>G	0.175	0.225	0.1	-0.125	0.125	0.35	-0.225	0.165	0.1675			0.079; 0.181
rs3208305	32067 A>T	0.35	0.3	0.225	-0.075	0.375	0.425	-0.05	0.335	0.3375			0.071; 0.319
rs1803924	32093 C>T	0.1	0.05	0.05	0	0.175	0.075	0.1	0.09	0.0913			0.024; 0.094
rs1059507	32382 C>T	0.175	0.2	0.1	-0.1	0.075	0.275	-0.2	0.165	0.1264	0.103		0.079; 0.181
rs3735964	32464 C>A	0.1	0.05	0.05	0	0.15	0.075	0.075	0.085	0.0903			0.024; 0.109
rs3200218	32490A>G	0.125	0.075	0.075	0	0.05	0.075	-0.025	0.08	0.1488			0.143; 0.217
rs13702	32911 T>C	0.35	0.3	0.225	-0.075	0.375	0.45	-0.075	0.34	0.3349	0.459	0.272	0.071; 0.319
**rs1059611**	32982 T>C	0.125	0.1	0.05	-0.05	0.2	0.075	0.125	0.115	0.1276		0.107	0.238; 0.109
rs3866471	33088 C>A	0.05	0.1	0	-0.1	0.05	0.15	-0.1	0.07	0.1615			
**rs15285**	33086 C>T	0.25	0.225	0.125	-0.1	0.3	0.25	0.05	0.23	0.3331			
	**3'Near**												
rs9644636	33315T>G	0.4	0.625	0.25	-0.375	0.35	0.475	-0.125	0.58	0.1793			
rs3916027	33287G>A	0.1	0.15	0	-0.15	0.125	0.15	-0.025	0.105	0.2895	0.413		
rs4921683	33487T>A	0.05	0.1	0	-0.1	0	0.125	-0.125	0.055	0.1268	0.102		
rs4921684	33547C>T	0.05	0.1	0	-0.1	0	0.125	-0.125	0.055	0.1178	0.079	0.149	

NLP: Kuwaiti samples with normal lipid levels; LTG: frequency of lower percentile of TG levels; HTG: frequency of Higher percentile of TG levels; HHDL: frequency of High percentile of HDL levels: LHD: frequency of low percentile of HDL levels; **TG∆F: difference of allele frequency between low and high TG groups; HDL∆F: difference of allele frequency between low and high HDL groups** KMAF: Kuwaiti Minor Allele Frequency; GMAF: Global Allele Frequency (Ensemble Browser; AA: African Americans; NHW: Non-hispanic Whites

Table H. Analysis of the distribution of variants in the introns at the *LPL* gene locus.

	Size (bp)	InDels	SNPs	Total	%*	Average site change**
Intron 1	8,651	5	53	58	26.48	149.16
Intron 2	3,428	3	23	26	11.87	131.85
Intron 3	1,361	0	8	8	3.65	170.13
Intron 4	698	2	2	4	1.83	174.50
Intron 5	1,487	0	17	17	7.76	87.47
Intron 6	3,176	8	29	37	16.89	85.84
Intron 7	1,520	3	16	19	8.68	80.00
Intron 8	1,031	3	10	13	5.94	79.31
Intron 9	3,090	5	32	37	16.89	83.51
**Total**		**29**	**190**	**219**		

* Percent of the variants in each intron from the total (n=219)

** Estimated based on the size of each intron and total number of variants observed.
